# Prolyl Hydroxylase Paralogs in *Nicotiana benthamiana* Show High Similarity With Regard to Substrate Specificity

**DOI:** 10.3389/fpls.2021.636597

**Published:** 2021-03-02

**Authors:** Réka Mócsai, Kathrin Göritzer, David Stenitzer, Daniel Maresch, Richard Strasser, Friedrich Altmann

**Affiliations:** ^1^Department of Chemistry, Institute of Biochemistry, University of Natural Resources and Life Sciences, Vienna, Austria; ^2^Department of Applied Genetics and Cell Biology, Institute of Plant Biotechnology and Cell Biology, University of Natural Resources and Life Sciences, Vienna, Austria

**Keywords:** prolyl-hydroxylase, *Nicotiana benthamiana*, substrate specificity, 4-hydroxyproline, plant-specific modification

## Abstract

Plant glycoproteins display a characteristic type of *O*-glycosylation where short arabinans or larger arabinogalactans are linked to hydroxyproline. The conversion of proline to 4-hydroxyproline is accomplished by prolyl-hydroxylases (P4Hs). Eleven putative *Nicotiana benthamiana* P4Hs, which fall in four homology groups, have been identified by homology searches using known *Arabidopsis thaliana* P4H sequences. One member of each of these groups has been expressed in insect cells using the baculovirus expression system and applied to synthetic peptides representing the *O*-glycosylated region of erythropoietin (EPO), IgA1, Art v 1 and the *Arabidopsis thaliana* glycoprotein STRUBBELIG. Unlike the situation in the moss *Physcomitrella patens*, where one particular P4H was mainly responsible for the oxidation of erythropoietin, the tobacco P4Hs exhibited rather similar activities, albeit with biased substrate preferences and preferred sites of oxidation. From a biotechnological viewpoint, this result means that silencing/knockout of a single *P4H* in *N. benthamiana* cannot be expected to result in the abolishment of the plant-specific oxidation of prolyl residues in a recombinant protein.

## Introduction

Plant-made pharmaceuticals are gaining more and more attention, therefore the difference between human post-translational modifications (PTMs) and plant PTMs should be addressed. One of the most prominent modifications to deal with is glycosylation of proteins. Glycoengineering of the *N*-glycosylation pathway of certain plants is already in a very advanced stage. Abolishment of the immunogenic plant-specific β1,2-xylose and α1,3-fucose residues of the core *N*-glycan was achieved in different plant systems (Koprivova et al., 2004; Strasser et al., 2004; Cox et al., 2006; Strasser et al., 2008; Sourrouille et al., 2008; Jansing et al., 2019) and production of erythropoietin (EPO) without Lewis-A epitopes was achieved in moss (Parsons et al., 2012). *O-*glycan engineering in plants on the other hand is still in its infancy and plant-produced pharmaceuticals with human-like structures devoid of plant-specific modifications have not been reported (Castilho et al., 2012; Yang et al., 2012). In mammals, *O-*glycan biosynthesis occurs in a stepwise fashion involving the sequential transfer of single monosaccharide residues to serine and threonine residues forming mucin-type *O*-glycans. A similar mucin-type *O-*glycan biosynthesis pathway does not exist in plants (Strasser, 2012). The first step in the plant-specific *O*-glycosylation is the generation of Hyp residues. Many Hyp residues are further glycosylated by arabinogalactan, extensin type oligo-arabinosyl glycan chains or solanaceous lectins (Clarke et al., 1979; Lamport, 1980; Showalter and Varner, 1989; Josè and Puigdomènech, 1993). Hyp residues are generated by peptidyl-proline 4-dioxygenase, also called prolyl 4-hydroxylase (P4H). P4Hs belong to the large class of non-heme iron dioxygenases that use α-ketoglutarate (2-oxoglutarate) and O_2_ as cosubstrates to activate the metal center and form succinate and a high-valent iron(IV)-oxo species (Price et al., 2003; Proshlyakov et al., 2004). These plant-specific modifications are undesired as concerns have been raised for the presence of non-human PTMs on recombinant proteins for therapeutic applications. Therefore, the responsible enzymes must be discovered and characterized in order to engineer *Nicotiana benthamiana* into a competitive expression system for recombinant *O-*glycosylated proteins.

In mammals two P4H families have been identified, Hypoxia Inducible Factor (HIF)-P4Hs and collagen-P4Hs. Hydroxylation of collagen proline residues results in the formation of the stable triple helical structure of collagen (Myllyharju, 2003). HIF-P4Hs regulate the stability of a transcription factor (HIF1a) (Epstein et al., 2001). Prolyl 4-hydroxylation can also be detected on other proteins like elastin (Müller et al., 1978) and even on the nuclear protein Argonaute 2 (Qi et al., 2008). Mammalian P4Hs have clear preference for the sequence X-Pro-Gly, typically Pro-Pro-Gly, whereby good substrate affinity is only provided by strings of more than five such triplets (Kivirikko et al., 1989). In plants, the motifs recognized by P4Hs are more ambiguous. Typical substrates are proline-rich glycoproteins and the subgroup of arabinogalactan proteins (AGPs), which occur in the extracellular space or are linked to the outer membrane of plant cells. An example from a tomato AGP is the sequence TGQTPAAAXVGAKAGTTPPAAP, where all prolines are converted to Hyp (Zhao et al., 2002). The synthetic sequences (SP)n, (SPP)n, and (SPPPP)n (fused to green fluorescent protein) were readily oxidized when expressed in *N. benthamiana* or *A. thaliana* (Xu et al., 2008). In the C-terminal sequence of Art v 1 (KSPPGATPAPPGAAPPPAAGGSPSPPADGGSPPPPADGGSPP VDGGSPPPPSTH) (Uniprot Q84ZX5), the major allergen of mugwort, prolines are hydroxylated to about 60% (Himly et al., 2003). In a ragweed allergen (Uniprot D4IIH8) Hyp residues are found in the sequence KNPGPPPGAPKGMPPAPSPPSGGGAPPPSGGE (Leonard et al., 2010). In BY-2 tobacco cells the hydroxylation of a single proline residue in the recombinant protein sporamin required a substrate with a five amino acid sequence [AVSTG]–Pro–[AVSTG]–[GAVPSTC]–[APSDE] where Pro is the modification site, while the other four amino acids can be either ones within the corresponding brackets (Shimizu et al., 2005). These examples indicate that plant P4Hs have a wider substrate specificity and/or many enzymes are involved in the oxidation of these proteins.

In *A. thaliana* as well as in *Solanum lycopersicum* 13 different P4H paralogs were reported so far (Hieta and Myllyharju, 2002; Tiainen et al., 2005; Kalaitzis et al., 2009; Velasquez et al., 2011). In the promising recombinant protein expression system *N. benthamiana* the P4H activity was already described (Pinkhasov et al., 2011; Castilho et al., 2012; Yang et al., 2012; Göritzer et al., 2017), but the putative P4Hs are still uncharacterized. Since this plant was already successfully used for production of therapeutic proteins for treatment of human patients (Qiu et al., 2014), the issue of proline hydroxylation should be addressed and examined further, to create strategies to eliminate this modification on plant-produced recombinant proteins.

In this work four *N. benthamiana* P4H candidates were selected from different homology groups and recombinantly expressed in insect cells using the baculovirus expression system. Enzyme activity was tested by four synthetic peptide substrates with specific focus on IgA1 proline-rich hinge region peptide. Subcellular localization was evaluated in plant leaf tissue as well as initial experiments were conducted for silencing of the genes coding for the two most active *P4H* candidates.

## Materials and Methods

### Plant Material and Growth Conditions

Wild-type *Nicotiana benthamiana* plants were grown under long-day conditions (16-h-light/8-h-dark photoperiod) on soil at 24°C.

### Selection of P4H Paralogs

To discover putative P4Hs in *N. benthamiana*, *Arabidopsis thaliana* DNA sequences of known *P4H* genes were used to perform BLAST (basic local alignment search tool) searches against the gene models in the *N. benthamiana* database^1^. Protein sequence alignments were performed using the MAFFT algorithm L-INS-i (Katoh and Standley, 2013). A maximum likelihood phylogenetic tree was produced using PhyML software (Guindon et al., 2010) at the web server NGPhylogeny.fr (Lemoine et al., 2019) using SPR moves to optimize tree topology and 10 random starting trees. Statistical branch support was calculated using 500 bootstrap replications and the optimal substitution model was assessed by Smart Model Selection (Lefort et al., 2017) to be GTR + G + I + F under the Akaike information criterion (AIC). Multiple sequence alignments and phylogenetic trees were visualized in the MEGA7 software (Kumar et al., 2016).

### P4H Cloning and Expression in Insect Cells

Total RNA was isolated from leaves of 5-week-old wild type *N. benthamiana* using the Monarch Total RNA Miniprep kit (New England Biolabs, Frankfurt am Main, Germany) and cDNA was prepared using the LunaScript RT SuperMix kit (New England Biolabs). Four selected candidates of each phylogenetically relevant groups were subcloned into NEB pMiniT 2.0 vectors provided with the NEB PCR mini kit (New England Biolabs). All primers are shown in Supplementary Table 1. Sequences were then verified by Sanger sequencing (Microsynth AG, Balgach, Switzerland) and are deposited in Supplementary Data 1. GenBank accession numbers were assigned to each of these nucleotides: MW524054 (Nb-P4H1), MW524055 (Nb-P4H4), MW524056 (Nb-P4H9) and MW524057 (Nb-P4H10). For expression in insect cells the catalytically active domain was amplified without the membrane anchor. The baculovirus expression system was used to multiply the pVT-Bac-His-1 vector (Tessier et al., 1991) carrying the *P4H* sequences in *Sf*9 cells, finally the obtained viral stocks were used to co-transfect *Trichoplusia ni* High five cells to express the recombinant P4H enzymes. The enzymes were His-tag purified from the cell supernatant with immobilized metal affinity chromatography using an Äkta purifier and 1 ml His-Trap FF columns (GE Healthcare Life Sciences, Vienna, Austria).

### P4H Activity Assay and LC-ESI-MS Measurements

The enzymatic assays to verify peptide hydroxylation were performed in a total of 50 μL 100 mM MES buffer (pH 6.3) containing 10 μL of purified enzyme, 100 μM synthetic peptide; 300 μM 2-oxoglutarate; 2 mM ascorbate; 50 μM FeSO_4_ for 16 h at 30°C. The exact amounts of the recombinant enzymes used were Nb-P4H1: 7.01 μg; Nb-P4H4: 8.25 μg; Nb-P4H9: 4.20 μg; Nb-P4H10: 7.26 μg as determined with the micro BCA protein assay kit (Thermo Fisher Scientific, Vienna, Austria). All the synthetic peptides were ordered from JPT (JPT Peptide Technologies, Berlin, Germany) and denatured by heating to 97°C for 10 min, followed by rapid cooling before using in the activity assays. The reaction mixtures were first passed through a 10mg Hypersep-96 C18 cartridge (Thermo Fisher Scientific) and eluted with 85% Acetonitrile in 80 mM Ammonium-formiate buffer (pH 3). The eluants were freeze-dried and taken up in starting buffer before being loaded onto a Biobasic capillary column (150 mm × 0.32 mm; Thermo Fisher Scientific) using a Dionex Ultimate 3000 LC-system (Thermo Fisher Scientific) directly coupled to a Bruker maXis 4G Q-TOF MS instrument equipped with the standard ESI source (Bruker Daltonics, Bremen, Germany). Spectra were recorded in positive ion data depended acquisition mode. The same setup was used for analyzing plant- and HEK293 cell expressed IgA1 samples that were transiently expressed in *N. benthamiana* and HEK293 cells as described in detail previously (Göritzer et al., 2017). Purified IgA1 samples first went through reduction, *S*-alkylation (iodoacetamide) and digestion with sequencing grade trypsin (Promega, Walldorf, Germany). For the relative quantification of the different hydroxyprolineated structures, peak areas of Extracted Ion Chromatograms (EICs) of the first four isotopic peaks were summed. All observed charge states were considered. In case of overlapping ammonium adducts the relative quantification was done similarly by exclusively considering the monoisotopic peaks of all observed charge states.

### Determination of K_*m*_ and v_*max*_ Values

To determine the K_*m*_ and v_*max*_ values the activity assays were downscaled to a final volume of 20 μL maintaining the same concentrations and temperature. The recombinant enzymes used were Nb-P4H1: 3.51 μg; Nb-P4H4: 1.65 μg; Nb-P4H9: 0.42 μg; Nb-P4H10: 1.45 μg per reaction. To calculate a K_*m*_ and a v_*max*_ value the concentration of the synthetic IgA1 peptide varied from 0.022 mM to 5.61 mM. For Nb-P4H1 and Nb-P4H4 a reaction time of 30 min was chosen, while for the other two enzymes the reaction was stopped after 20 min. The assays were immediately purified with 25 mg Hypersep-96 C18 cartridges (Thermo Fisher Scientific) using 85% Acetonitrile in 80 mM Ammonium-formiate buffer (pH 3) to elute the peptides. For MS measurements the peptides were either loaded onto a Biobasic capillary column (150 mm × 0.32 mm; Thermo Fisher Scientific) or onto an Acclaim PepMap nano column (250 mm × 0.075 mm; Thermo Fisher Scientific) using a Dionex Ultimate 3000 LC system (Thermo Fisher Scientific) coupled to a Bruker maXis 4G Q-TOF MS with the standard or the nano ESI source (Bruker Daltonics). To assist relative quantification, a fixed amount of another synthetic peptide (PTTTPITTTTTVTPTPTPTGTQTK) was added to each sample as internal standard. To quantify the hydroxylated products, it was assumed that the relation between the standard and the substrate is the same as the relation between the standard and the product.

### RNAi-Mediated Transient Silencing of Endogenous *Nb-P4H1* and *Nb-P4H10* Genes

For RNAi-mediated gene silencing of endogenous *Nb-P4H1* and *Nb-P4H10*, a sense-intron-antisense construct was generated following a previously established procedure (Strasser et al., 2008). A subcloned synthetic DNA fragment consisting of a *Nb-P4H1* or *Nb-P4H10* sense DNA fragment fused to the intron 2 from the *Arabidopsis thaliana* β1,2-xylosyltransferase gene (Supplementary Data 2) was obtained from GeneArt Gene Synthesis (Thermo Fisher Scientific). The synthetic DNA was used as a template for PCR amplification of the corresponding antisense fragment using the primers listed in Supplementary Table 1. The antisense PCR fragment was *Kpn*I/*Bam*HI digested and ligated into the respective cloning vector carrying the sense-intron DNA. The sense-intron-antisense sequence was subsequently excised from the cloning vector and ligated into the *Xba*I/*Bam*HI digested plant expression vector pPT2M (Strasser et al., 2005).

The pEAQ-IgA1 light and heavy chain expression vectors were available from a previous study (Göritzer et al., 2017). IgA1 constructs were additionally cloned into the *Xba*I/*Bam*HI sites of pPT2M (Strasser et al., 2005) after amplification of the light and heavy chain constructs from the previously described GeneArt String fragments (Göritzer et al., 2017). In the pPT2M vector the expression is under the control of a cauliflower mosaic virus 35S promoter.

For transient gene silencing, overnight cultures of agrobacteria carrying the expression vector for the IgA1 heavy chain (either pEAQ-IgA1-HC or pPT2M-IgA1-HC) were diluted to an optical density (OD600) of 0.15, bacteria carrying the IgA1 light chain (pEAQ-IgA1-LC or pPT2M-IgA1-LC) or the gene silencing constructs were diluted to an OD600 of 0.1. Equal volumes were mixed and the mixture was infiltrated into *N. benthamiana* wild-type plants as described previously (Strasser et al., 2008). Two (pPT2M-constructs) or 4 days (pEAQ-constructs) after infiltration, IgA1 was purified from leaves as described previously (Göritzer et al., 2017) and subjected to LC-ESI-MS analysis. To determine the gene silencing efficiency, leaves of 5-week-old *N. benthamiana* wild-type plants were infiltrated with the *Nb-P4H1*-RNAi or *Nb-P4H10*-RNAi silencing constructs. Infiltration with pPT2M-IgA1-HC was used as a mock control. RNA was isolated 2-days after infiltration from 25 mg leaves using the Monarch Total RNA Miniprep Kit (New England Biolabs). Total RNA was reverse transcribed using the LunaScript RT SuperMix Kit (New England Biolabs). qPCR was performed in triplicate using the GoTaq qPCR Master Mix (Promega) and the CFX96 Touch Real-Time PCR System (Bio-Rad, Feldkirchen, Germany). Protein phosphatase 2A (PP2A) expression was used for normalization of qPCR data. Data analysis was done with the CFX-Manager software version 3.1 (Bio-Rad). Three independent biological replicates were used to calculate the mean values and standard deviation.

### Subcellular Localization

For Nb-P4H1-RFP expression, the full-length *Nb-P4H1* coding region was amplified from the subcloned *Nb-P4H1* pMiniT 2.0 clone with the primers listed in Supplementary Table 1, *Xba*I/*Bam*HI digested and cloned into *Xba*I/*Bam*HI digested expression vector p31 (Hüttner et al., 2012). The Nb-P4H4-GFP expression construct was generated by PCR amplification from the NEB pMiniT 2.0 clone, *Xba*I/*Bgl*II digestion and ligation into *Xba*I/*Bam*HI digested expression vector p47 (Hüttner et al., 2014). *Nb-P4H9* and *Nb-P4H10* were cloned in a similar manner into p47. All constructs were transformed into *Agrobacterium tumefaciens* strain UIA143 and used for syringe-mediated agroinfiltration (Strasser et al., 2008). Leaves of 5-week-old wild type *N. benthamiana* plants were infiltrated with *Agrobacterium* suspensions carrying the protein(s) of interest with an OD600 of 0.1. Confocal images were acquired 2 days post infiltration (dpi) on a Leica SP5 confocal microscope (Leica Microsystems, Wetzlar, Germany). Samples were excited using 488- and 561-nm laser lines for GFP and RFP, respectively, and observed using a 63 × /1.4 NA or 100 × /1.4 NA CS2 STED white oil immersion objective, respectively. Signals were collected simultaneously from 500 to 530 nm for GFP and 600–630 nm for RFP. Post-acquisition image processing was performed in Adobe Photoshop CS6 (Adobe Systems Incorporated, San Jose, CA, United States). For co-localization RFP/GFP-tagged P4Hs were co-expressed with the *Cis*/medial-Golgi marker AtGnTI-RFP or AtGnTI-GFP (Schoberer et al., 2019).

### Transient Nb-P4H Overexpression in *Nicotiana benthamiana* Leaves

Agrobacteria carrying one of the Nb-P4H-GFP or -RFP fusion constructs were diluted to an OD600 of 0.1 and mixed with agrobacteria carrying pEAQ-IgA1-HC (OD600 of 0.15) and agrobacteria carrying pEAQ-IgA1-LC (OD600 of 0.1). The mixture was infiltrated into leaves of 5-week old wild-type *N. benthamiana* using syringe-mediated agroinfiltration (Strasser et al., 2008). 4 days after infiltration, IgA1 was purified from leaves as described in detail previously (Göritzer et al., 2017) and subjected to LC-ESI-MS analysis.

## Results

### Identification of Prolyl 4-Hydroxylases in *Nicotiana benthamiana*

**FIGURE 1:**
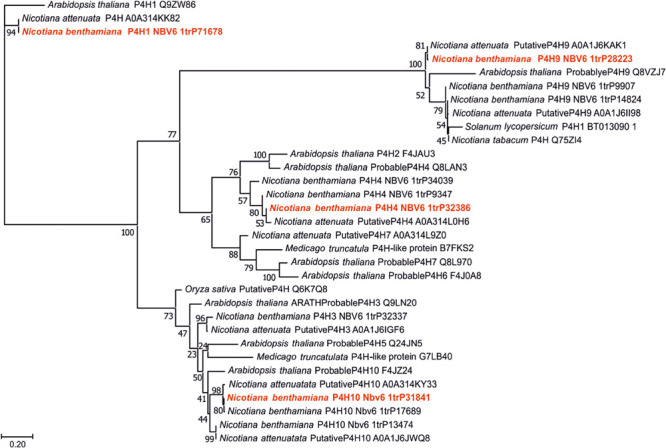
Phylogeny of the catalytic domains of different putative or known prolyl 4-hydroxylases (P4Hs). All putative *N. benthamiana* P4Hs were compared with relevant plant enzymes. Sequences are derived from UniProt (UniProt accession numbers are given for all entries) and version 6 of *N. benthamiana* database (www.uniprot.org; benthgenome.qut.edu.au). Protein sequences were aligned using the MAFFT alignment tool with the algorithm L-INS-i (Katoh and Standley, 2013) and phylogeny was inferred using PhyML (Guindon et al., 2010). Branch support was calculated by 500 bootstrap repetitions (values displayed in percent). The tree was visualized in MEGA7 (Kumar et al., 2016). For further experiments the selected candidates are marked red.

For the identification of P4H paralogs in *N. benthamiana*, the nucleic acid sequences of *Arabidopsis thaliana P4H*s were used to perform BLAST searches against the gene models in the *Nicotiana benthamiana* database (see text footnote 1) (Supplementary Table 2). With this approach, 11 putative *P4H*s could be determined. A phylogenetic analysis was carried out including protein sequences of these *N. benthamiana P4H* candidates and other *P4H* sequences of different plant origin. Only the putative catalytical domains were aligned to minimize algorithm errors. Some of the known plant *P4H*s (eg., *P. patens, C. reinhardtii*, and *C. sorokiniana*) displayed very low sequence identity to *N. benthamiana P4H* candidates, therefore these sequences were omitted from the final alignment. The resulting tree depicted four clearly separating branches (Figure 1). The catalytically active domains of one paralog from each group were selected and successfully amplified from *N. benthamiana* cDNA libraries. The candidates were chosen based on the expression levels in the leaf tissue (Supplementary Table 3), these data were obtained from the Gene Expression Atlas (version 6) of the *N. benthamiana* database (see text footnote 1). The selected *Nb-P4H* candidate genes were the following: Nbv6.1TRP71678 (*Nb-P4H1*), Nbv6.1TRP32386 (*Nb-P4H4*), Nbv6.1TRP28223 (*Nb-P4H9*), and Nbv6.1TRP31841 (*Nb-P4H10*). Interestingly, aligning several biological replicates of amplified and cloned sequences of *Nb-P4H4* and *Nb-P4H10* with the sequences of the *N. benthamiana* database showed some discrepancies in sequence identities (Supplementary Table 4). This potentially represents natural differences in *N. benthamiana* accessions (Schiavinato et al., 2019). However, this did not disrupt enzyme activity probably attributed to the fact that the amino acids in key positions for binding cofactors and substrate were unaffected by these mutations. Supplementary Figure 1 shows the amino acid sequence alignment of the obtained sequences next to the *A. thaliana* P4H1 sequence. As clearly marked, the three Fe^2+^-binding residues (two histidines and an aspartate), and the lysine binding the C-5 carboxyl group of the 2-oxoglutarate are conserved for all Nb-P4H candidates.

**FIGURE 2:**
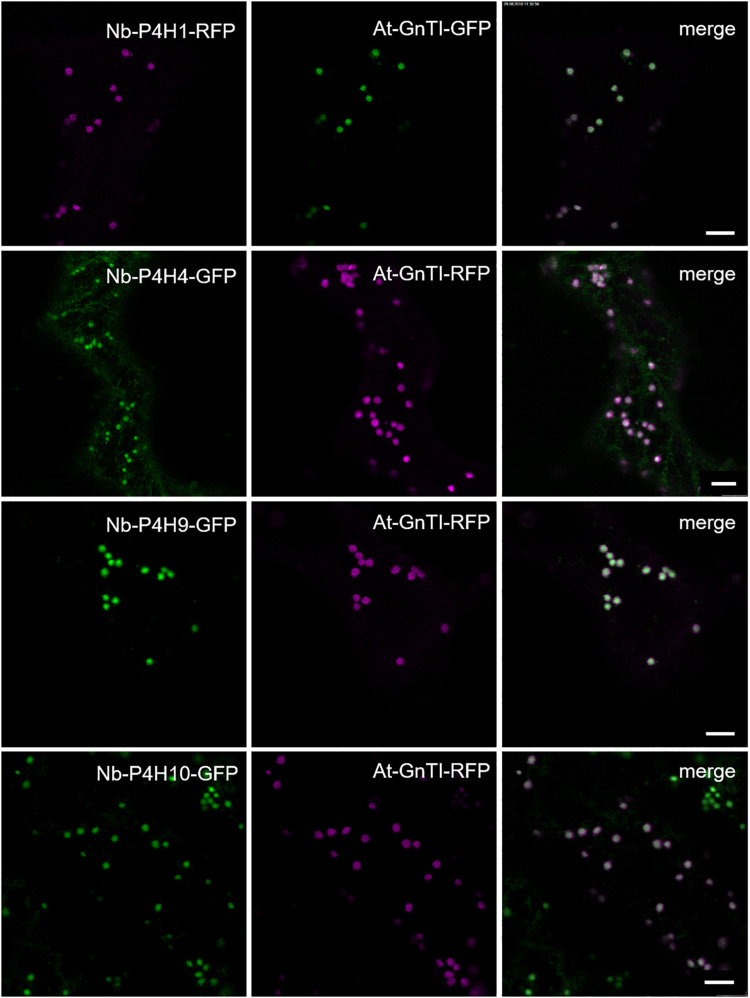
Subcellular localization of potential *Nicotiana benthamiana* P4Hs fused with fluorescent proteins. Co-localization was performed with *Cis*-Golgi located *Arabidopsis thaliana N*-acetylglucosaminyltransferase I (GnTI). Scale bars = 10 μm.

To confirm the expression and monitor the subcellular localization of the Nb-P4H proteins, they were transiently expressed in *N. benthamiana* leaves with a fluorescent protein fused to the C-terminus. Co-localization experiments with the *Cis*/medial-Golgi resident *N*-acetylglucosaminyltransferase I (GnTI) (Schoberer et al., 2019) showed that all Nb-P4H candidates were located in the Golgi apparatus (Figure 2). In addition to the Golgi localization, Nb-P4H4 was also present in the ER. Overall, the localization of the Nb-P4H candidates is consistent with a previous study that detected a tobacco P4H in the ER and Golgi in tobacco BY2 cells (Yuasa et al., 2005).

### Enzyme Activity and Substrate Specificity of Recombinant Nb-P4Hs

The DNA sequences coding for the catalytic domains from all 4 *Nb-P4H*s were cloned into a baculovirus expression vector and expressed in insect cells. Prolyl 4-hydroxylase activity was assessed for each recombinant enzyme by mass spectrometry using synthetic peptides designed based on experiments conducted previously. These peptides had the following four sequences: VTVPVPSTPPTPSPSTPPTPSPS from IgA1 and AQKEAISPPDAASAA from EPO, representing the critical parts of two biotechnologically relevant naturally *O-*glycosylated human proteins, AAGGSPSPPADGGSPPPPADG from the arabinan and arabinogalactan carrying *Artemisia vulgaris* pollen allergen Art v 1 (Leonard et al., 2005) and DGTPFNTSIITPPPPPCCDPPPATHR from the *Arabidopsis thaliana* protein STRUBBELIG (Vaddepalli et al., 2011).

**FIGURE 3:**
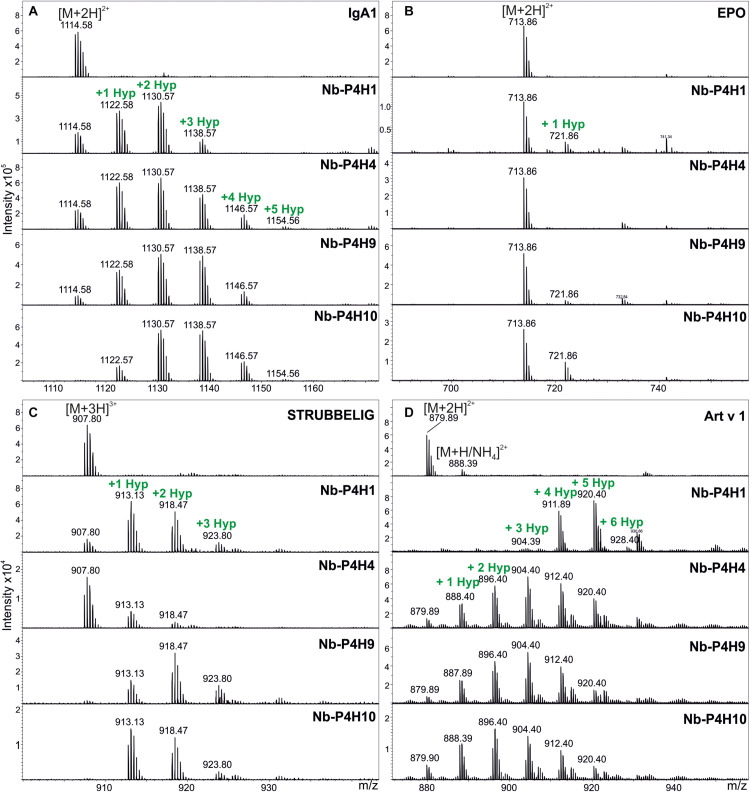
Activity of recombinant Nb-P4Hs on different synthetic peptide substrates. **(A)** Representative ESI-LC-MS spectra of doubly charged ions [(M + 2H)^2+^] of the IgA1 peptide (VTVPVPSTPPTPSPSTPPTPSPS), **(B)** double charged ions of the EPO peptide (AQKEAISPPDAASAA), **(C)** triple charged ions [(M + 3H)^3+^] of the STRUBBELIG peptide (DGTPFNTSIITPPPPPCCDPPPATHR), **(D)** double charged ions of the Art v 1 peptide (AAGGSPSPPADGGSPPPPADG). Proline hydroxylation can be observed by the mass increments of 16 Da in single charged ions (or 8 and 5.3 Da in double and triple charged ions, respectively). The isotopic pattern is correct for each peptide except for Art v 1 where an undesired ammonium adduct (+17 Da) adds to the intensity of the second isotopic peak. In this case the height of the first isotopic peak should be accepted.

**FIGURE 4:**
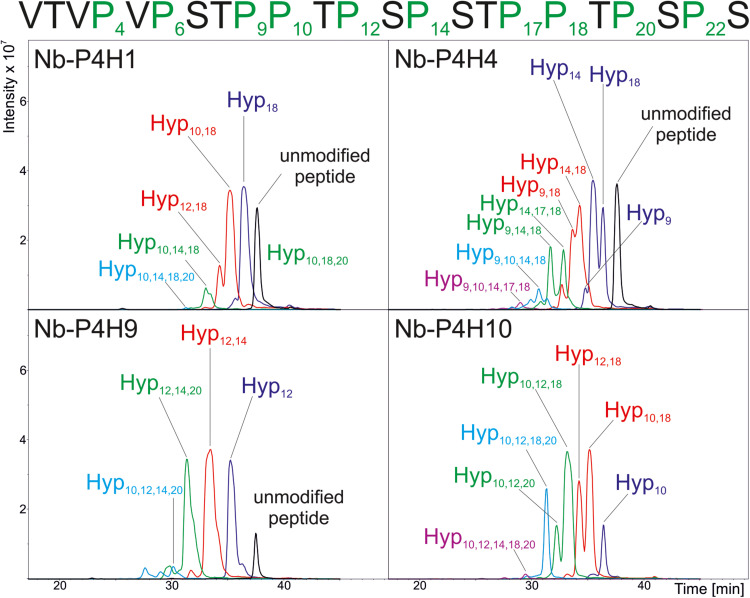
Summarized results of the site-specific analysis of P4H activity of recombinant P4H candidates from *N. benthamiana.* Base Peak Chromatograms of the LC-ESI-MS runs are depicted and location of oxidation sites are marked as detected by MS/MS analysis of the synthetic IgA1 sequence VTVPVPSTPPTPSPSTPPTPSPS. Individual MS^2^ spectra for each peak can be found in Supplementary Figure 5.

Using the IgA1 peptide, K_*m*_ and v_*max*_ values were determined by mass spectrometry (Table 1 and Supplementary Figures 2, 3, 4). Although using a very specific substrate and utilizing a novel method for determination of these values make the comparison difficult, our results fit in well with the published results of other plant P4Hs (Eriksson et al., 1999; Hieta and Myllyharju, 2002; Tiainen et al., 2005; Keskiaho et al., 2007). At first glance, all four recombinant P4Hs from *N. benthamiana* displayed similar activity and substrate specificity (Figure 3). Each enzyme consistently oxidized up to five Pro residues of the IgA1 peptide during overnight assays, and they all acted on all other synthetic peptides with the exception of Nb-P4H4 on EPO. In the EPO peptide, there are two prolines present that are next to each other but only one of them (Pro_149_) was oxidized by Nb-P4H1, Nb-P4H9, and Nb-P410. Nb-P4H1 was the most active enzyme on the extension-like sequence of Art v 1, while Nb-P4H9 and Nb-P4H10 preferred mainly the STRUBBELIG and IgA1 sequences. The cofactors 2-oxoglutarate, ascorbate and Fe^2+^ were all essential to the activity of these enzymes, concluded from inactivity in the absence of these substances (data not shown). A closer inspection by MS/MS, however, revealed clear differences of the four P4Hs in site and substrate preferences (Figure 4 and Supplementary Figure 5). The MS fragmentation data showed that each enzyme – despite having broad substrate specificity – shows a preference for specific sites. The preferred sequence of recombinant Nb-P4H1 as well as Nb-P4H10 is P**P**TPS in which the second proline gets oxidized (marked bold). Interestingly, this sequence occurs repeatedly in the IgA1 hinge-region peptide and while Nb-P4H1 mostly acts on P_18_, Nb-P4H10 preferably oxidizes P_10_. Furthermore, Nb-P4H4 displays activity on the same site as Nb-P4H1 but mostly prefers the sequence S**P**STP. Nb-P4H9 on the other hand was most active at the site T**P**SPS. Surprisingly, the prolines at the sites V**P**VPS, V**P**STP and the C terminal S**P**S harboring P_4_, P_6_, and P_22_ residues of the IgA1 sequence were seemingly untouched by any of the recombinant enzymes tested. A total of 7 Hyp residues could be identified as target substrates of the IgA1 peptide, indicating that only 70% of prolines are oxidized by these enzymes. Lastly, Nb-P4H activity was probed with HEK293-cell produced IgA1 substrates as well, however, no conversion of proline to hydroxyproline could be detected (data not shown) possibly caused by different folding or the occupancy of the nearby *O-*glycosylation sites.

**TABLE 1:** Michaelis–Menten constant (K_*m*_) and maximum velocity (v_*max*_) values of recombinant *N. benthamiana* P4Hs using the synthetic peptide from IgA1 as substrate (VTVPVPSTPPTPSPSTPPTPSPS).

Recombinant enzyme	K_*m*_ (μM)	v_*max*_ (μM/min)
Nb-P4H1	8	0.03
Nb-P4H4	1900	0.1
Nb-P4H9	72	0.4
Nb-P4H10	66	0.02

*K_*m*_ and v_*max*_ values were calculated based on Hanes-Woolf plots Supplementary Figure 4, panel B1-B4.*

### Overexpression of Nb-P4H1, Nb-P4H4, Nb-P4H9, and Nb-P4H10 Fusion Proteins and RNAi-Mediated Transient Silencing of *Nb-P4H1* and *Nb-P4H10* Genes

**FIGURE 5:**
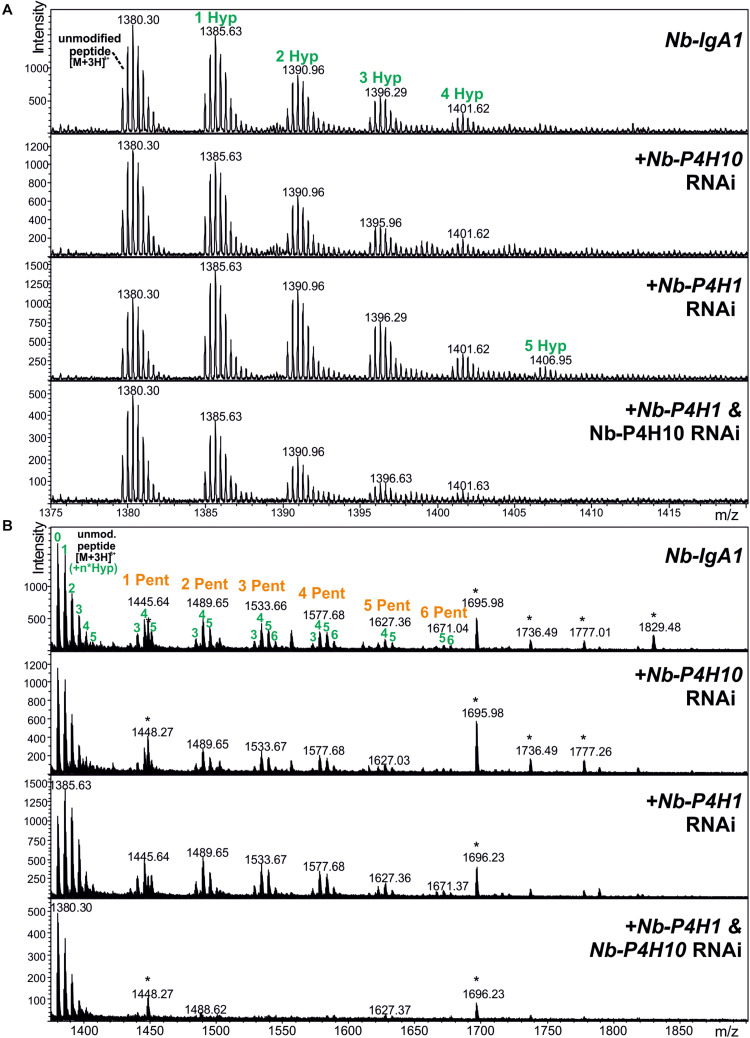
LC-ESI-MS spectra of the hinge region peptide from recombinant IgA1 produced in the presence of *Nb-P4H* silencing constructs (*Nb-P4H1* RNAi and *Nb-P4H10* RNAi). Recombinant IgA1 was expressed by infiltration of pPT2M-IgA1. **(A)** Unmodified peptide and additional peaks showing P4H activity. **(B)** A wider mass range shows the same samples carrying additional *O*-glycans in the form of arabinosylation. Asterisks denote impurities unrelated to the target molecules.

Monomeric IgA1 was co-expressed with different fluorescent protein-tagged Nb-P4Hs to see whether their overexpression results in increased Hyp formation in the IgA1 hinge region. Recombinant IgA1 was purified from crude leaf extract, digested with trypsin and applied to ESI-LC-MS analysis to investigate the changes in proline hydroxylation on the IgA1 hinge region. Relative quantification by peak integration revealed highest degrees of hydroxylation through co-expression with Nb-P4H1-RFP and Nb-P4H10-GFP (Table 2A and Supplementary Figure 6). We therefore generated *Nb-P4H1* and *Nb-P4H10* RNAi silencing constructs for co-infiltration with IgA1 expression vectors in *N. benthamiana*. The used RNAi construct is based on a design that we successfully used previously in stable or transient silencing approaches and gives good expression 2 to 3 days after infiltration (Strasser et al., 2008; Shin et al., 2017). In addition to the pEAQ-IgA1 expression vector that shows highest expression 4 days after infiltration (Göritzer et al., 2017), IgA1 was expressed using the same binary expression vector (pPT2M) that was used for expression of the gene silencing constructs and gives maximal expression levels 2 to 3 days after infiltration. By co-infiltration of pPT2M-IgA1 with *Nb-P4H1*/*Nb-P4H10*-RNAi the highest percentage of non-modified IgA1 hinge-region could be reached (Figure 5). Even though the proline modification was reduced, keeping over 50% of the peptides unmodified, the Nb-P4H activity could not be completely abolished by only silencing two of the *Nb-P4H*s (Table 2B). This is in agreement with the observed site-specificity of different recombinant Nb-P4H on synthetic substrates suggesting that several enzyme paralogs are active on a given peptide substrate in *N. benthamiana*. On the other hand, a surprising feature of *Nb-P4H1* and *Nb-P4H10* silencing was the greatly reduced attachment of pentoses to the remaining Hyp residues (Figure 5).

**TABLE 2:** Relative quantification of hydroxyproline residues in the hinge-region tryptic peptide (HYTNPSQDVTVPCPVPSTPPTPSPSTPPTPSPSCCHPR – 4136.8899 Da) of plant produced IgA1 co-infiltrated with *Nb-P4H* overexpression and silencing constructs.

A		Hyp residues							av. #Hyp	% change
		0	1	2	3	4	5	6		
pEAQ-IgA1	*Nb-IgA1*	26.3	27.9	23.9	16.0	4.8	1.1	0.1	1.49	100
	+*Nb-P4H1* overexpression	9.6	12.5	17.2	25.4	25.5	8.0	1.7	2.75	195
	+*Nb-P4H4* overexpression	32.0	28.4	19.9	13.7	4.8	1.2	0.1	1.35	90
	+*Nb-P4H9* overexpression	20.7	23.5	21.8	20.9	9.7	2.9	0.3	1.85	127
	+*Nb-P4H10* overexpression	18.0	23.1	27.4	19.6	8.3	3.1	0.5	1.88	130

**B**		**Hyp residues**							**av. #Hyp**	**% change**
		**0**	**1**	**2**	**3**	**4**	**5**	**6**		

pEAQ-IgA1	*Nb-IgA1*	26.3	27.9	23.9	16.0	4.8	1.1	0.1	1.49	100
	+*Nb-P4H10*-RNAi	35.2	31.0	19.7	6.6	4.0	1.9	1.7	1.26	83
	+*Nb-P4H1* and *P4H10*-RNAi	31.5	31.0	20.5	10.8	4.2	1.4	0.7	1.32	87
pPT2M-IgA1	*Nb-IgA1*	32.2	30.7	18.8	11.4	5.2	1.5	0.3	1.33	100
	+*Nb-P4H1*-RNAi	23.1	30.5	23.6	14.4	6.1	1.9	0.4	1.57	119
	+*Nb-P4H10*-RNAi	36.9	31.5	17.1	8.9	4.1	1.3	0.2	1.17	88
	+*Nb-P4H1* and *P4H10*-RNAi	50.5	27.0	13.7	6.0	2.5	0.4	0.0	0.84	64

*Panel **(A)** includes the results showing the effect of overexpression of all 4 Nb-P4H proteins (fused to RFP or GFP) on hydroxyproline formation. Panel **(B)** shows relative quantification results using silencing constructs for *Nb-P4H1* and *Nb-P4H10*. The two different expression vectors (pPT2M and pEAQ, which display maximal expression at different time points) for recombinant production of IgA1 are evaluated separately. Values are presented in percent (%) using the summed peak areas of all observed charge states. The first four isotopic peaks were considered for each of the charge states; in case of ammonium adduct formation only the monoisotopic peak was considered. The average number of Hyp residues per peptide is listed below (av. #Hyp) together with percent increase or decrease (%change) compared to the wild IgA1 construct (*Nb-IgA1*). The corresponding mass spectra can be found in Supplementary Figure 6 and in Figure 5.*

## Discussion

In addition to already well-established mammalian cell systems, the plant-based production of recombinant immunoglobulins is gaining more and more attention as plants allow the production of customized homogeneous *N*-glycans with few engineering steps (Strasser et al., 2014; Loos et al., 2014). The next step to tailor immunoglobulin’s posttranslational glycosylation is to direct our attention toward *O*-glycans and the elimination of plant-specific modifications. These start with a proline 4-hydroxylase oxidizing a proline residue in the substrate polypeptide chain. This is then followed by glycosyltransferases building up arabinans or arabinogalactans (Leonard et al., 2005; Leonard et al., 2010). Arabinose residues attached to *O-*glycosylation sites have been described for maize-produced IgA and for a mucin-tandem repeat containing peptide expressed in *N. benthamiana* (Karnoup et al., 2005; Pinkhasov et al., 2011). These plant-specific glycans could cause immunogenic or allergic reactions in humans (Leonard et al., 2005). Engineering mucin-type *O-*glycans onto MUC1 was successful in *N. benthamiana*, but the unsubstituted hydroxyprolines were still a concern in the work of Yang et al., 2012.

Even sialylated structures could be engineered into *N. benthamiana* without causing phenotype changes, losing biomass or any adverse effect on expression yield (Castilho et al., 2012). Still, hydroxyproline remained integrated in the product, which asks for further engineering steps. In this work four different homology groups of potential P4Hs from *N. benthamiana* were identified. Members of each of these groups, that were assumed as potentially relevant for heterologous protein production were expressed and characterized. Consistent with previous data on BY2 cells (Yuasa et al., 2005) or transient expression of Arabidopsis P4Hs in *N. benthamiana* (Velasquez et al., 2015), all selected Nb-P4H candidates were localized in the Golgi apparatus, with Nb-P4H4 showing presence in the ER as well.

Our selected method to evaluate P4H activity was LC-ESI-MS. Prolyl 4-hydroxylation is clearly detectable by searching the MS spectra for additional peaks with the mass increment of 16 Da. Four synthetically produced peptides, two from human proteins of biomedical interest and two from plant proteins were used as substrate for the recombinant enzymes. Differences in substrate preferences were observed, but in general all 4 enzymes were active on all four substrates with the exception of Nb-P4H4 on human erythropoietin peptide. Moreover, LC-ESI-MS/MS also revealed remarkable differences in the order of hydroxylation of different proline residues within a substrate. Eventually, although in different progression, all recombinant Nb-P4Hs oxidized the same target sequences on our substrates, showing no differences in specificity. Human IgA1 peptide was used as substrate for developing a new method to obtain the Michaelis-Menten constants and values of the reactions’ maximum velocity. These values didn’t show greater differences between the different *N. benthamiana* enzymes than those reported in *A. thaliana* P4H orthologs (Tiainen et al., 2005). Based on our results, Nb-P4H1 (K_*m*_ = 8 μM) and Nb-P4H10 (K_*m*_ = 66 μM) have the highest affinity to bind to the IgA1 hinge region peptide, which is in agreement with the overexpression results.

Substrate specificity of plant P4Hs is still not well defined and poly-L-proline was accepted as a general substrate (Tanaka et al., 1980). Initially, this suggested the recognition of the characteristic three-residue-per-turn extended helix, a conformation rather than a sequence. However, Hyp and glycosylated Hyp occur on sequences lacking this polyproline II conformation (Kieliszewski and Lamport, 1994), which implies that plant P4Hs, just like animal P4Hs, are indeed essentially directed by amino acid sequence. In green algae, contiguous Pro residues, Pro residues alternating with some other amino acid, mostly Ser, and PPSPX repeats have been identified in hydroxyproline-rich glycoproteins (where X means any amino acid), with many of the Pro residues being subsequently 4-hydroxylated (Adair and Snell, 1990; Sumper and Hallmann, 1998; Ferris et al., 2001). The *Chlamydomonas reinhardtii* P4H1 also used SPKPP as substrate amongst other motifs already mentioned above, albeit with lesser efficiency (Keskiaho et al., 2007). This is a rare occurrence, because Pro followed by a Lys residue is not hydroxylated in higher plants but is a good substrate of a viral P4H (Kieliszewski and Lamport, 1994; Eriksson et al., 1999). In BY-2 tobacco cells the 5 amino acid sequence required for proline 4-hydroxylation was described as [AVSTG]–**P**–[AVSTG]–[GAVPSTC]–[APSDE] (modification site marked bold) (Shimizu et al., 2005). Our findings showed that all investigated Nb-P4Hs used the following 5 sequence motif in the IgA1 peptide: [VSTP]–**P**–[STP]–[TP]–[SP]. This complements the sequence requirements of Shimizu et al. (2005) with Pro residues in both +1 and −1 position to the modification site. In a tomato AGP, all prolines of the peptide sequence TGQTPAAAXVGAKAGTTPPAAP were converted to Hyp, creating similar motifs with prolines in both +1 and −1 positions (Zhao et al., 2002). With the help of the EPO peptide, another motif (PPDAA) was revealed to be substrates of some Nb-P4Hs. Nb-P4H1, Nb-P4H9 and Nb-P4H10 utilized this motif where Asp is in +1 position of the hydroxylation site. This motif was also discovered as substrate for moss P4H1 (Parsons et al., 2013). We proved that Nb-P4Hs are acting on various different substrates and new hydroxylation sites were discovered. It is clear that the sequence requirements are far from specific. This might cause issues with not only IgA and EPO, but at the production of other glycoproteins as well. There is a clear need for further understanding and successful elimination of P4H activity in order to utilize *N. benthamiana* as a safe host for the expression of recombinant glycoproteins.

By overexpression of the selected candidates in *N. benthamiana* leaves, some candidates seemed to act on the IgA1 proline-rich peptide more, therefore silencing constructs were developed for transient co-expression with glycoproteins. Using this approach, the quantity of Hyp residues and the attached pentoses on the plant produced recombinant IgA1 were perceivably reduced. Pentoses, presumably arabinoses (Shpak et al., 2001; Xu et al., 2008), were only observed where three or more Hyp residues were present. The exact arrangement of these substrates may be relevant for arabinosyl transferases, which would explain the stronger reduction of glycosylation compared to that of hydroxylation. Glycosylation was previously shown to have more rigid sequence requirements compared to hydroxylation (Shimizu et al., 2005). However, the removal of these modifications was not complete, which may be explained by insufficient reduction of mRNA levels (30% reduction for *Nb-P4H1 and* 85% reduction for *Nb-P4H10*, Supplementary Figure 7) or a sizable expression of other members of the homology group. Based on the sequence identity to *P4H* candidates in the *N. benthamiana* genome database (Supplementary Table 2 and Supplementary Data 2) it can be predicted that the used *Nb-P4H1*-RNAi construct is quite specific for *Nb-P4H1*. The *Nb-P4H10*-RNAi sequence, on the other hand, displays high sequence identity to other *Nb-P4H10* and *Nb-P4H3* candidates that we did not characterize in this study. It is therefore plausible that the silencing affects also other members of the homology group, which could be an advantage for the prevention of unwanted Hyp formation on recombinant proteins. Stable expression of the gene silencing constructs or a complete knockout of *Nb-P4H* genes may completely prevent Hyp formation. In addition, it might be necessary to characterize and eliminate other enzyme paralogs that are active on a given recombinant glycoprotein expressed in *N. benthamiana*.

Obviously, any strategy toward elimination of proline 4-hydroxylation in *N. benthamiana* will have to consider the possibility of phenotypic changes. The effect of P4H action on plant phenotype depends on the particular P4H and its substrate. Elimination of AtP4H2, AtP4H5 and AtP4H13 in *Arabidopsis* resulted in a short root hair phenotype (Velasquez et al., 2011; Velasquez et al., 2015). In *Arabidopsis* a correct *O-*glycosylation on extensins seemed essential for cell-wall assembly and, hence, root hair elongation. In the moss *P. patens* the knockout of single *P4H*s did not compromise the viability of moss cells (Parsons et al., 2013). A profound effect on general plant growth was revealed by gene silencing of putative *P4H*s in tomato (Fragkostefanakis et al., 2014). Interestingly, silencing of three different *P4H*s in tomato resulted in an increased leaf area due to enhanced cell division and cell expansion. While the complete elimination could potentially have adverse side effects, an increased leaf area would also be favorable in terms of biomass for recombinant protein production in *N. benthamiana*. Strategies to overcome potential adverse effects of Nb-P4H elimination include the use of powerful transient gene silencing or tissue or even cell-type specific genome editing approaches (Huang et al., 2009; Wang et al., 2020). Moreover, a better understanding of the substrate specificities of different P4Hs may allow the generation of engineered P4H variants that act specifically on endogenous plant proteins without hydroxylating recombinant human proteins. The here characterized Nb-P4Hs and their elimination are first steps toward the development of an expression system for recombinant glycoproteins with custom-made *O-*glycans lacking Hyp and associated plant-specific glycosylation.

## Data Availability Statement

Publicly available datasets were analyzed in this study. This data can be found here: *Nicotiana benthamiana* database (https://benthgenome.qut.edu.au/).

## Author Contributions

RM was involved in most experiments, analysis, and interpretation of the data as well as drafting the manuscript. KG planned and executed plant expression and silencing experiments. DS did the enzyme kinetic experiments. DM provided insights and help with mass spectrometry and data quantification. FA and RS conceived and supervised the work. All authors have revised and approved the final version.

## Conflict of Interest

The authors declare that the research was conducted in the absence of any commercial or financial relationships that could be construed as a potential conflict of interest.

## Footnotes

1 https://benthgenome.qut.edu.au/
