# Supplementary material for: Prolyl Hydroxylase Paralogs in Nicotiana benthamiana Show High Similarity With Regard to Substrate Specificity
Source: Front Plant Sci. 2021 Mar 2;12:636597. doi: 10.3389/fpls.2021.636597 (PMC7960765; doi:10.3389/fpls.2021.636597)
Supplement: Supplementary file 1 [file Data_Sheet_1.pdf]

Supplementary Material

to

Prolyl hydroxylase paralogs in *Nicotiana benthamiana* show high similarity with regard to substrate specificity

Réka Mócsai[1], Kathrin Göritzer[2], David Stenitzer[1], Daniel Maresch[1], Richard Strasser[2], Friedrich Altmann[1*]

[1] Institute of Biochemistry, Department of Chemistry, University of Natural Resources and Life Sciences, Vienna, Austria

[2] Institute of Plant Biotechnology and Cell Biology, Department of Applied Genetics and Cell Biology, University of Natural Resources and Life Sciences, Vienna, Austria

*** Correspondence:**
friedrich.altmann@boku.ac.at

SUPPLEMENTARY TABLES

Supplementary Table S1. All oligonucleotides used in this study are collected in this table.

	Construct	F 5'→3'	R 5'→3'	Size (bp)
Subcloning vector pMiniT	*Nb_P4H1*	TCCTCCATTTCAGTTCAGATTTC	GGACAAAAATGTTCTAAGATATATCAA	961
	Nb_P4H4	AGGAACTTATCTCTATCTCTTCTGTCA	AGTGGGAGGGAGAGGTATAATG	1000
	Nb_P4H9	CCTCTCTATTTGGGTTGTGTTGT	AGAAGGGACAAACTGATACTGAGC	1102
	Nb_P4H10	CAGCGACTCAGACTTCATACACT	GGCTCTCCTCAAGATTGTAACC	1077
Expression vector pVT-Bac-His-1	*Nb_P4H1*	GTTTCTGAGCTCCATAGGATGGAGGACTCACTTGAC	GTTTCTGGTACCTTAGGATACAAGTCTTTGCCTCATCC	771
	Nb_P4H4	GTTTCTGAGCTCAACCCATCCAAAGTCAAGCAAAT	GTTTCTGGTACCTTAACAGGCTTTGCAGCTCTTCC	801
	Nb_P4H9	GTTTCTGAGCTCGGTTCTACTCTCATCTCTCAGCAGG	GTTTCTGGTACCTTATTCATCAAGTTCCTGATCCCTG	771
	Nb_P4H10	GTTTCTGAGCTCTCGATTCCTTTCAGTTCTAAAGG	GTTTCTGGTACCTTAAACCTTGTATTCGTGAACACG	747
Plant expression vectors (GFP/RFP)	*Nb_P4H1*	TATATCTAGAATGGCTTCGGCAATGAGAATTGTT	TATAGGATCCGGATACAAGTCTTTGCCTCATCC	858
	Nb_P4H4	TATATCTAGAATGAACAGCTCTTTGCTGCTCG	TATAAGATCTACAGGCTTTGCAGCTCTTCC	882
	Nb_P4H9	TATATCTAGAATGAAGAACAGAGGCAAATTAC	TATAGGATCCTTCATCAAGTTCCTGATCCCTG	867
	Nb_P4H10	TATATCTAGAATGGCAGTCAAAGGAAGGCACGTC	TATAGGATCCAACCTTGTATTCGTGAACACGCATC	870
RNAi vectors	*Nb_P4H1*	TATAGGTACCTTGCCTCATCCATTTAGTAG	TATAGGATCCGAGGGGGGAGAGACATACTTTC	213
	Nb_P4H10	TATAGGTACCGCATCCATTTCGTAGACGACC	TATAGGATCCAAATGGTGGTCAACGCATTGCC	278
RT-qPCR	*Nb-P4H1*	GGACTCACTTGACAGAGAATCG	TGCTAACGGATGTTTGTTGC	73
	Nb-P4H10	GGAACCAAGAGCTGTTGTGT	CCCCTGGCAAGAAATGTTCC	168
	Nb-PP2A	GACCCTGATGTTGATGTTCGCT	GAGGGATTTGAAGAGAGATTTC	123

Supplementary Table S2. Summarized results of the BLAST searches against *Nicotiana benthamiana* sequence database (https://benthgenome.qut.edu.au/) using all known *Arabidopsis thaliana* prolyl 4-hydroxylases (gene accession numbers in brackets). The candidates represented four phylogenetically distant groups, as shown in **Figure 1**. The last three hits were omitted from the list of candidates based on lack of transmembrane domains (TMD) and low homology scores. The 11 candidates were then phylogenetically assessed and 4 of them were selected for further characterization (marked red).

#	Name	closest to	score	Comment
1	**Nbv6.1trP31841 probable P4H 10**	*At-P4H3* (At1g20270)	1e-58	**Cloned**
2	**Nbv6.1trP13474 probable P4H 10**	*At-P4H10* (At5g66060)	1e-55	84% identity to #1
3	**Nbv6.1trP17689 probable P4H 10**	*At-P4H3* (At1g20270)	4e-49	96% identity to #1
4	**Nbv6.1trP32337 probable P4H 3**	*At-P4H3* (At1g20270)	2e-29	79% identity to #1
5	**Nbv6.1trP71678 prolyl 4-hydroxylase 1**	*At-P4H1* (At2g43080)	8e-23	**Cloned**
6	**Nbv6.1trP28223 probable P4H 9**	*At-P4H9* (At4g33910)	3e-16	**Cloned**
7	**Nbv6.1trP14824 probable P4H 9**	*At-P4H9* (At4g33910)	4e-15	81% identity to #6
8	**Nbv6.1trP32386 probable P4H 4**	*At-P4H4* (At5g18900)	3e-13	**Cloned**
9	**Nbv6.1trP9347 probable P4H 4**	*At-P4H4* (At5g18900)	3e-13	92% identity to #8
10	**Nbv6.1trP34039 probable P4H 4**	*At-P4H4* (At5g18900)	2e-11	83% identity to #8
11	**Nbv6.1trP9907 probable P4H 9**	*At-P4H9* (At4g33910)	6e-08	93% identity to #6
12	Nbv6.1trP27956 probable 28s rRNA (cytosine-c)-methyltransferase isoform x1	*At P4H7* (At3g28480)	1e-06	P4H domain, no TMD
13	Nbv6.1trP61995 hmg1 2-like protein	*At-P4H13* (At2g23096)	0.057	low homology, no P4H domain
14	Nbv6.1trP17158 probable uncharacterized protein	*At-P4H12* (At4g25600)	0.060	low homology, no P4H domain

Supplementary Table S3. RNA expression levels of the 11 identified prolyl 4-hydroxylases in *Nicotiana benthamiana* leaf tissue. Data were obtained from the Gene Expression Atlas (version 6) in the *N. benthamiana* database (https://benthgenome.qut.edu.au/) The candidates selected for cloning and expression are marked bold. In two cases, data was not available (NA).

**Transcript ID**	**Name**	**Expression level in leaf tissue (reads per million)**
**Nbv6.1trP71678**	**prolyl 4-hydroxylase 1**	5
**Nbv6.1trP32386**	**probable prolyl 4-hydroxylase 4**	32
Nbv61trP34039	probable prolyl 4-hydroxylase 4	20
Nbv61trP9347	probable prolyl 4-hydroxylase 4	13
**Nbv6.1trP28223**	**probable prolyl 4-hydroxylase 9**	37
Nbv61trP14824	probable prolyl 4-hydroxylase 9	4
Nbv61trP9907	probable prolyl 4-hydroxylase 9	NA
**Nbv6.1trP31841**	**probable prolyl 4-hydroxylase 10**	14
Nbv61trP17689	probable prolyl 4-hydroxylase 10	8
Nbv61trP13474	probable prolyl 4-hydroxylase 10	5
Nbv61trP32337	probable prolyl 4-hydroxylase 3	NA

Supplementary Table S4.

Comparison of nucleic acid sequences of the *N. benthamiana* database (https://benthgenome.qut.edu.au/) entries (template) and sequences of the selected clones acquired after amplification from *N. benthamiana* cDNA library.

**Template**	**Clone**	**Query cover(%)**	**Identity (%)**	**Mutations**	**Gaps**
Nbv6.1trP71678	*Nb-P4H1*	100	100	0	0
Nbv6.1trP32386	*Nb-P4H4*	100	99.63	3	0
Nbv6.1trP28223	*Nb-P4H9*	100	100	0	0
Nbv6.1trP31841	*Nb-P4H10*	100	95.60	30	3

SUPPLEMENTARY FIGURES

```
CLUSTAL O(1.2.4) multiple sequence alignment


Arath_P4H1_Q9ZW86      VSWSPRIIVLHDFLSPEECEYLKAIARPRLQVSTVVDVKTG-KGVKSDVRTSSGMFLTHV     59
NbP4H-1                ISWKPRIILFHNFLSAEECDYLRSVAMPRLHVSTVVDAKTG-KGIKSDVRTSSGMFLSPD     59
NbP4H-4                ISWKPRAFVYEGFLTDEECNHLISLAKSELKRSAVADNESG-NSKTSEVRTSSGMFIPKA     59
NbP4H-9                LSWFPRALYFPNFATEEQCQGIIKMAKAELKPSALALRKGETAENTKGIRTSSGMFISSS     60
NbP4H-10               ISWEPRAVVYHNFLSKDECEYLINLGKPHMKKSTVVDSATG-KSTDSRVRTSSGTFLARG     59
                       :** ** .   .* : ::*: :  :.  .:: *::.          . :***** *:

                                                                ↓ ↓
Arath_P4H1_Q9ZW86      ERSYPIIQAIEKRIAVFSQVPAENGELIQVLRYEPQQFYKPHHDYFADTFNLKRGGQRVA     119
NbP4H-1                ERKYPMIQAIEKRISVYSQIPVENGELIQVLRYEKNQFYRAHHDYFSDSFNVKRGGQRIA     119
NbP4H-4                --KDPIVSGIEEKIATWTFLPKENGEEIQVLRYEEGQKYEPHYDYFVDEVNIARGGHRLA     117
NbP4H-9                EDKTGILDLIEEKIARAAMIPRTHGEAFNVLRYEIGQSYHSHYDAFDPSQYGPQKSQRVA     120
NbP4H-10               --QDKVVRTIEKRIADFTFIPVEHGEGLQILHYEVGQKYEPHYDYFAEEFNTINGGQRIA     117
                         .  ::  **::*:  : :*  :** :::*:**  * *. *:* *       . .:*:*

Arath_P4H1_Q9ZW86      TMLMYLTDDVEGGETYFPLAGDGDC--------TCGGKIMKGISVKPTKGDAVLFWSMGL     171
NbP4H-1                TMLMYLSDGVEGGETYFPMAGTGEC--------SCGGKMIKGLCVKPTKGDAVLFWSMGL     171
NbP4H-4                TVLMYLTDVEKGGETVFPNAEESPRRRSMTADDSLSECAKKGIPVKPRKGDALLFYSLHP     177
NbP4H-9                SFLLYLSDVEEGGETMFPFENGQNMDANY------DFRKCIGLKVKPRRGDGLLFYSLFP     174
NbP4H-10               TVLMYLSDVEEGGETVFPTAKGNVS--AVPWWNELSECGKGGLSVKPKMGDALLFWSMKP     175
                       :.*:**:*  :**** **                 .     *: ***  **.:**:*:

                                ↓      ↓
Arath_P4H1_Q9ZW86      DGQSDPRSIHGGCEVLSGEKWSATKWMRQKA  202
NbP4H-1                DGQSDPESLHGGCEVLSGEKWSATKWMRQRL  202
NbP4H-4                NATPDPLSLHGGCPVIQGEKWSATKWIHVDS  208
NbP4H-9                NGTIDPTSLHGSCPVIRGEKWVATKWIR---  202
NbP4H-10               DATLDPSSLHGGCPVIKGNKWSSTKWMRVHE  206
                       :.  **  *:**.* *: *:** :***::
```

Supplementary Figure S1. Alignment of the catalytic domain sequences of the selected *N. benthamiana* P4H candidates next to the *Arabidopsis thaliana* P4H1 (Uniprot Q9ZW86). Arrows mark the three Fe^{2+}-binding residues (two histidines and an aspartate), and the lysine binding the C-5 carboxyl group of the 2-oxoglutarate. Alignment was created with the Clustal Omega Multiple sequence alignment tool (Madeira et al., 2019).

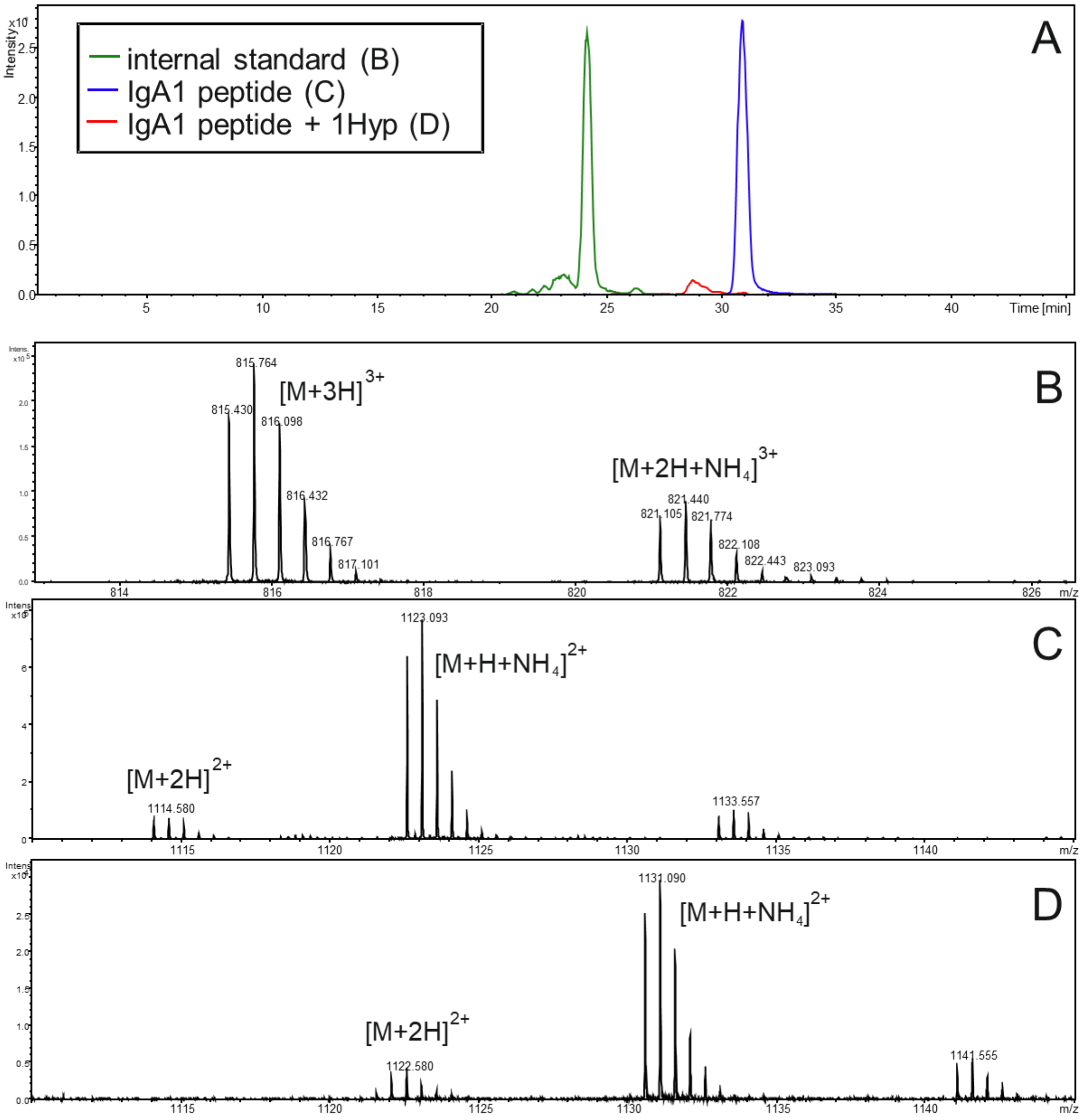

Supplementary Figure S2. A capillary-LC-ESI-MS measurement example (Nb-P4H9; 1.12 mM substrate). Assays were taken up in water and loaded onto a Biobasic capillary column (150×0.32 mm Thermo Scientific) using a Dionex Ultimate 3000 LC system coupled to a Bruker maXis 4G Q-TOF MS equipped with the standard ESI source. The concentration of the internal standard (PTTTPITTTTTVTPTPTPTGTQTK) remained the same for all measurements and was used for normalization of peak areas. The peak areas of the **(A)** base peak chromatograms were used for calculation. Representative mass spectra are depicted of the **(B)** internal standard, **(C)** the substrate IgA1 synthetic peptide (VTVPVPSTPPTPSPSTPPTPSPS) and the **(D)** one times oxidized product. Due to eluent composition the IgA1 peptide dominantly occurs as ammonium adduct, hence this form was used for quantification.

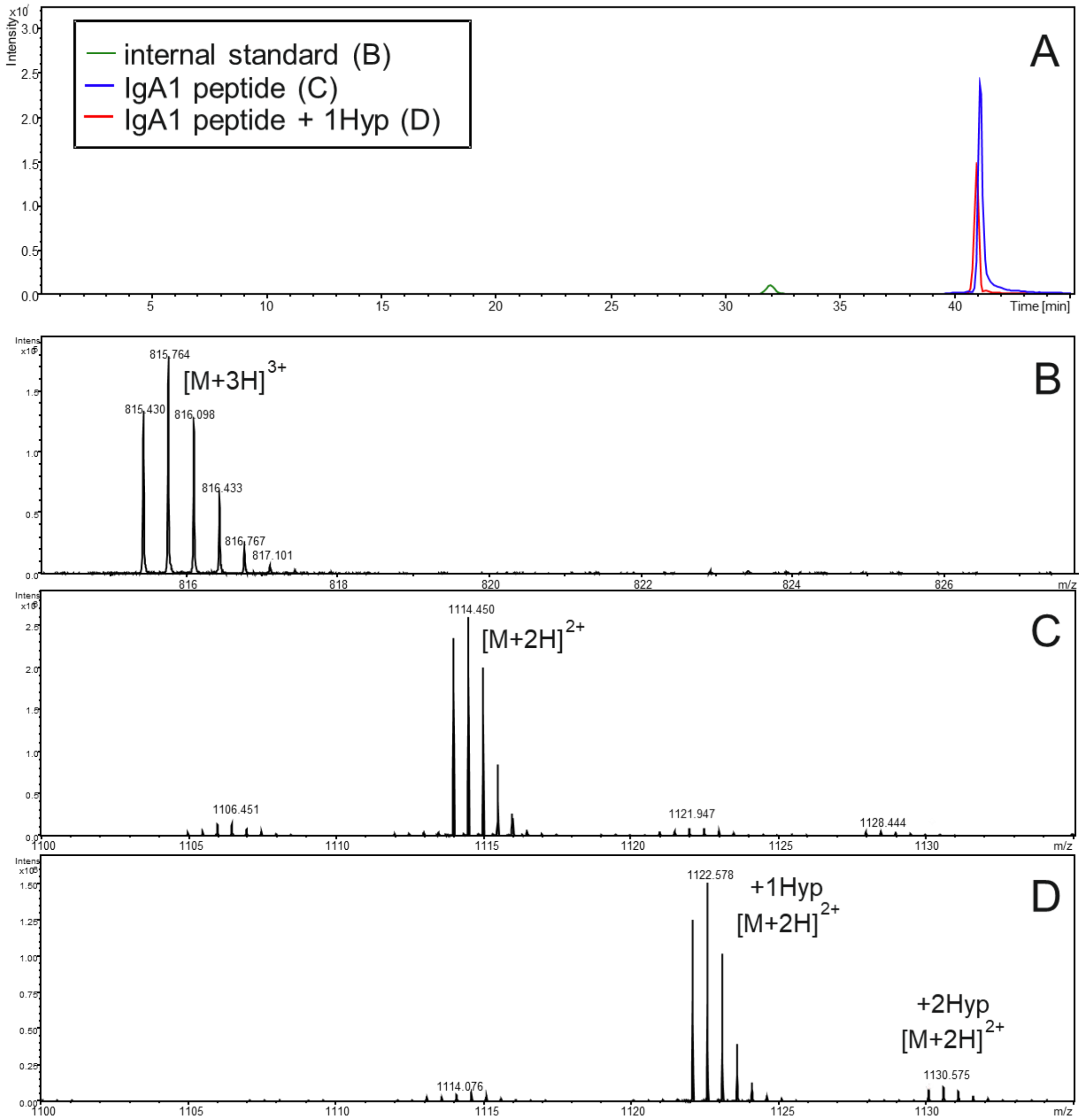

Supplementary Figure S3. A nano-LC-ESI-MS measurement example (Nb-P4H4; 1.12 mM substrate). Assays were taken up in water and loaded onto a Thermo Acclaim PepMap300 RSLC C18 separation column (2 µm particle size, 150*0.075 mm) with a Thermo Acclaim PepMap µ-precolumn using a Dionex Ultimate 3000 LC system coupled to a Bruker maXis 4G Q-TOF MS equipped with the nano ESI source. The concentration of the internal standard (PTTTPITTTTTVTPTPTPTGTQTK) remained the same for all measurements and was used for normalization of peak areas. The peak areas of the **(A)** base peak chromatograms were used for calculation. Representative mass spectra are depicted of the **(B)** internal standard, **(C)** the substrate IgA1 synthetic peptide (VTVPVPSTPPTPSPSTPPTPSPS) and the **(D)** one times oxidized product. In the product spectra also a two times oxidized peptide (+2Hyp) can be found. Due to the negligible amount it was excluded from the calculations.

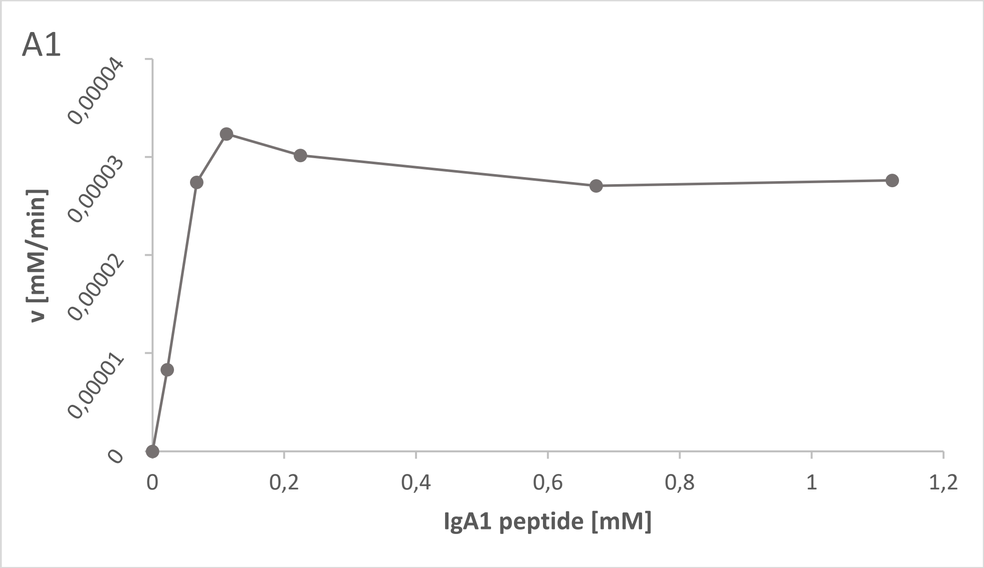

A1
v [mM/min]
0
0,00001
0,00002
0,00003
0,00004
0
0,2
0,4
0,6
0,8
1
1,2
IgA1 peptide [mM]

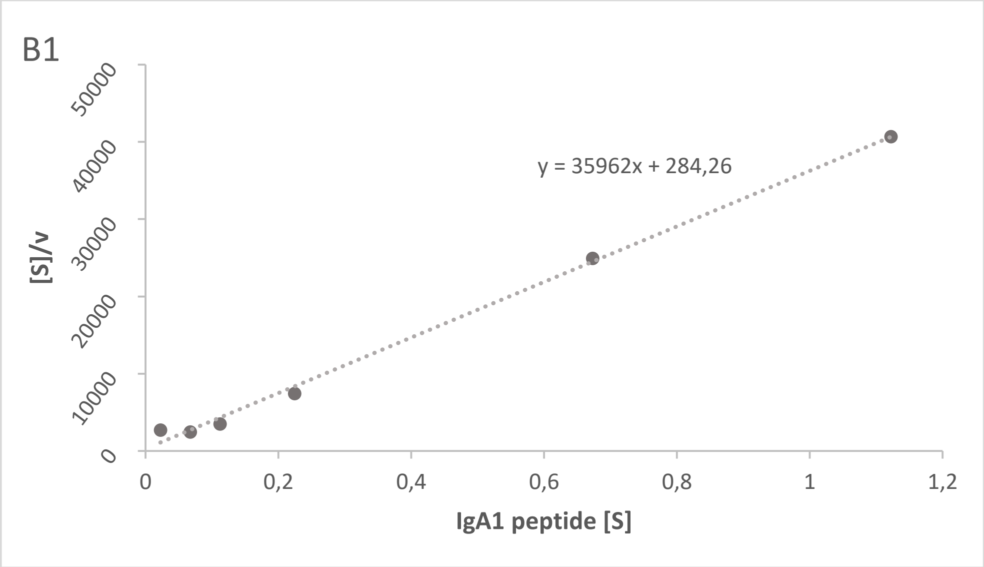

B1
[S]/v
0
10000
20000
30000
40000
50000
y = 35962x + 284,26
0
0,2
0,4
0,6
0,8
1
1,2
IgA1 peptide [S]

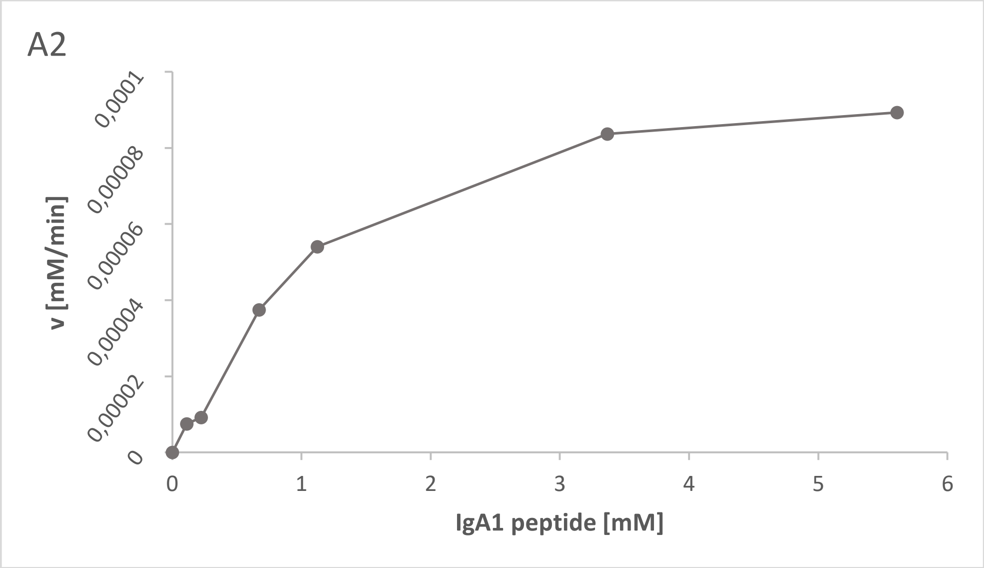

A2
v [mM/min]
0
0,00002
0,00004
0,00006
0,00008
0,0001
0
1
2
3
4
5
6
IgA1 peptide [mM]

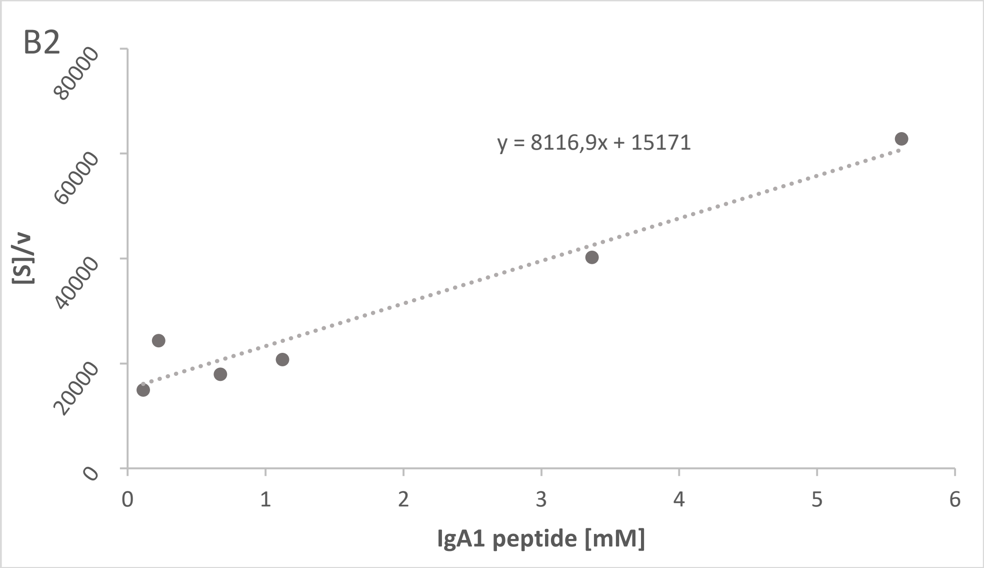
B2
[S]/v
80000
60000
40000
20000
0
y = 8116,9x + 15171
0
1
2
3
4
5
6
IgA1 peptide [mM]

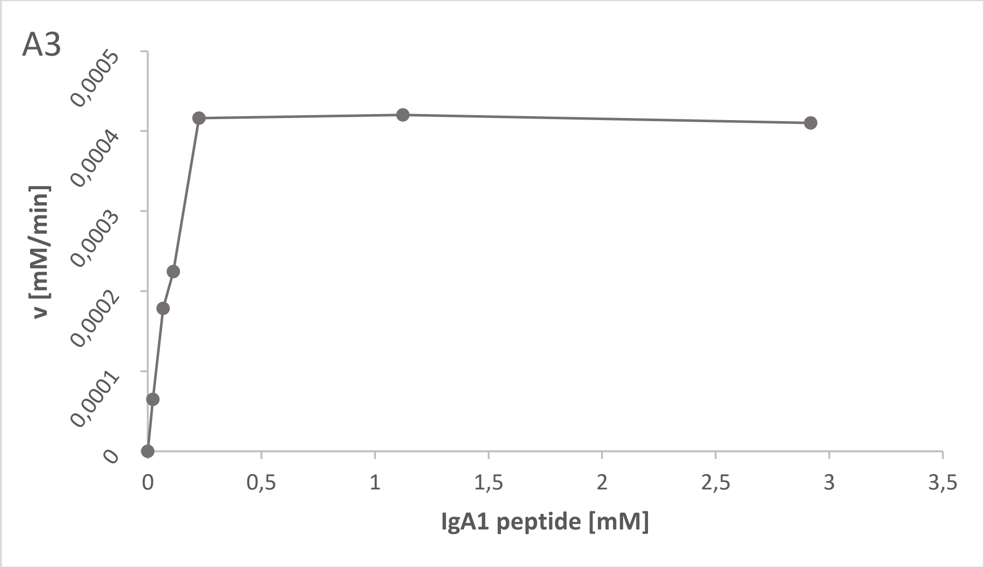
A3
v [mM/min]
0,0005
0,0004
0,0003
0,0002
0,0001
0
0
0,5
1
1,5
2
2,5
3
3,5
IgA1 peptide [mM]

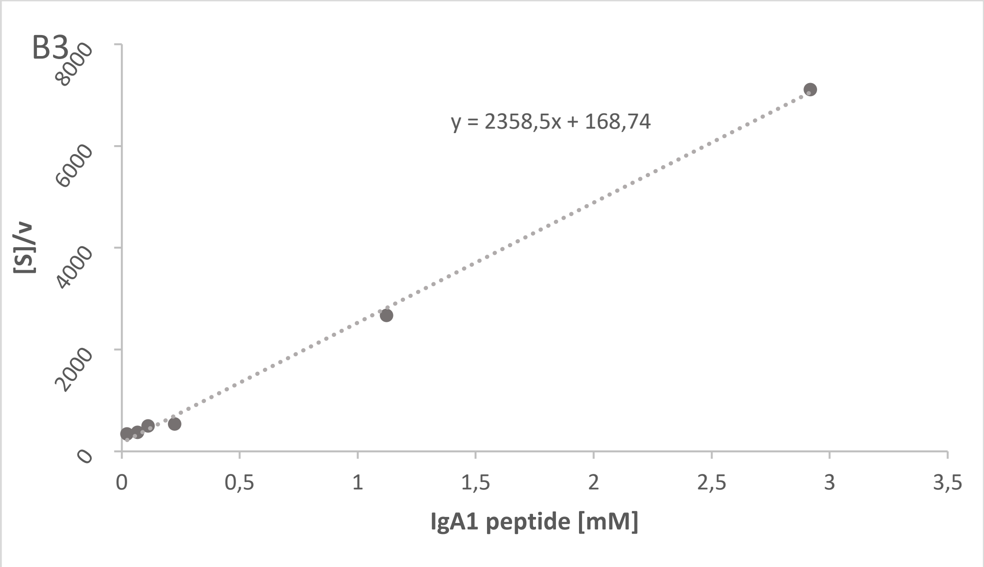
B3
[S]/v
8000
6000
4000
2000
0
y = 2358,5x + 168,74
0
0,5
1
1,5
2
2,5
3
3,5
IgA1 peptide [mM]

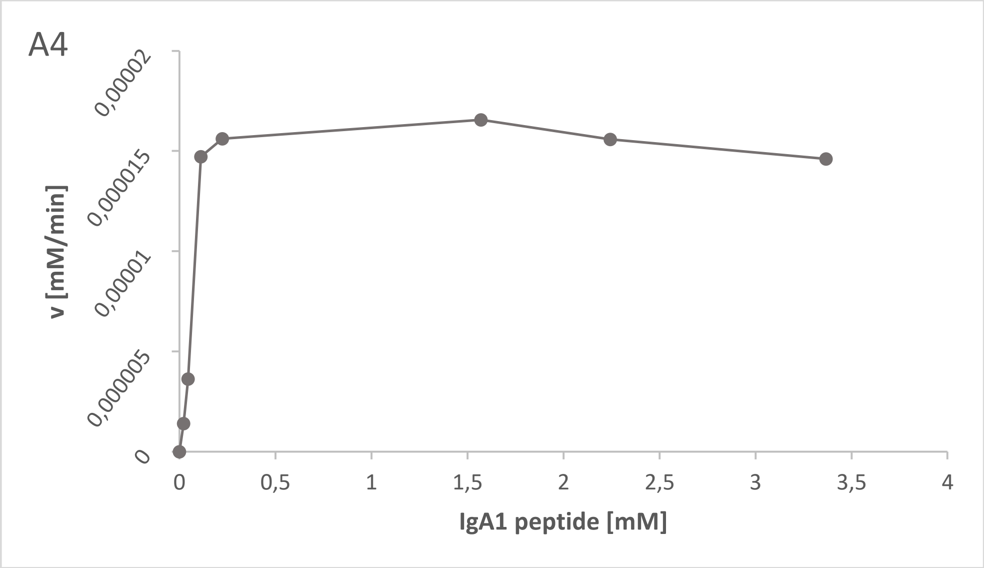

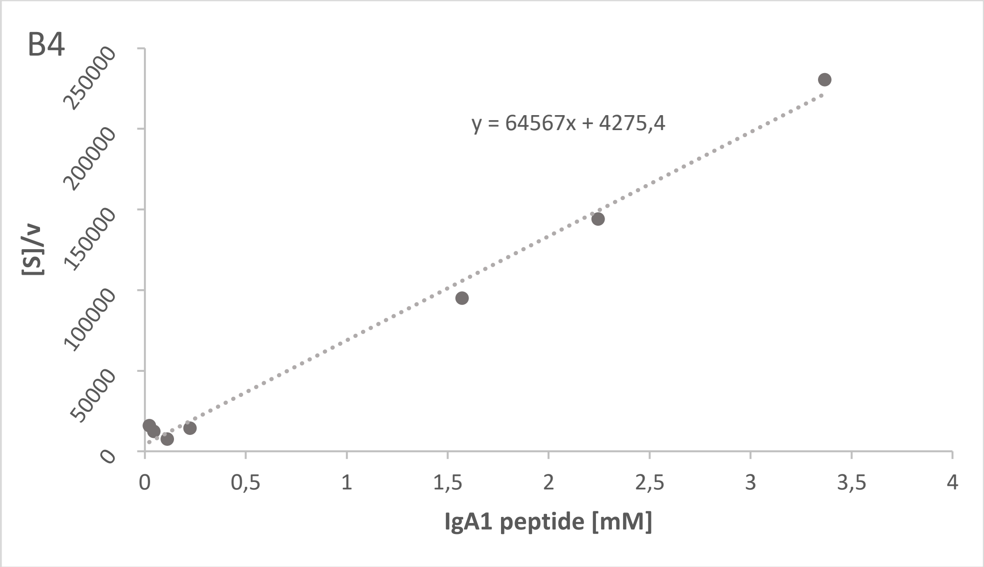

Supplementary Figure S4. K_m and v_{max} values were determined for **(A1+B1)** Nb-P4H1, **(A2+B2)** Nb-P4H4, **(A3+B3)** Nb-P4H9 and **(A4+B4)** Nb-P4H10. Concentrations from 0.02 to 5.61 mM of synthetic IgA1 peptide and a reaction time of 20 minutes (Nb-P4H9 and Nb-P4H10) or 30 minutes (Nb-P4H1 and Nb-P4H4) were used to calculate the K_m and v_{max} value. Analysis of the assays was done by LC-ESI-MS. **(A1-A4)** shows the velocity, which was calculated using a standard peptide, plotted against the substrate concentration. **(B1-B4)** Hanes-Woolf Plots for the calculation of the K_m and v_{max} values.

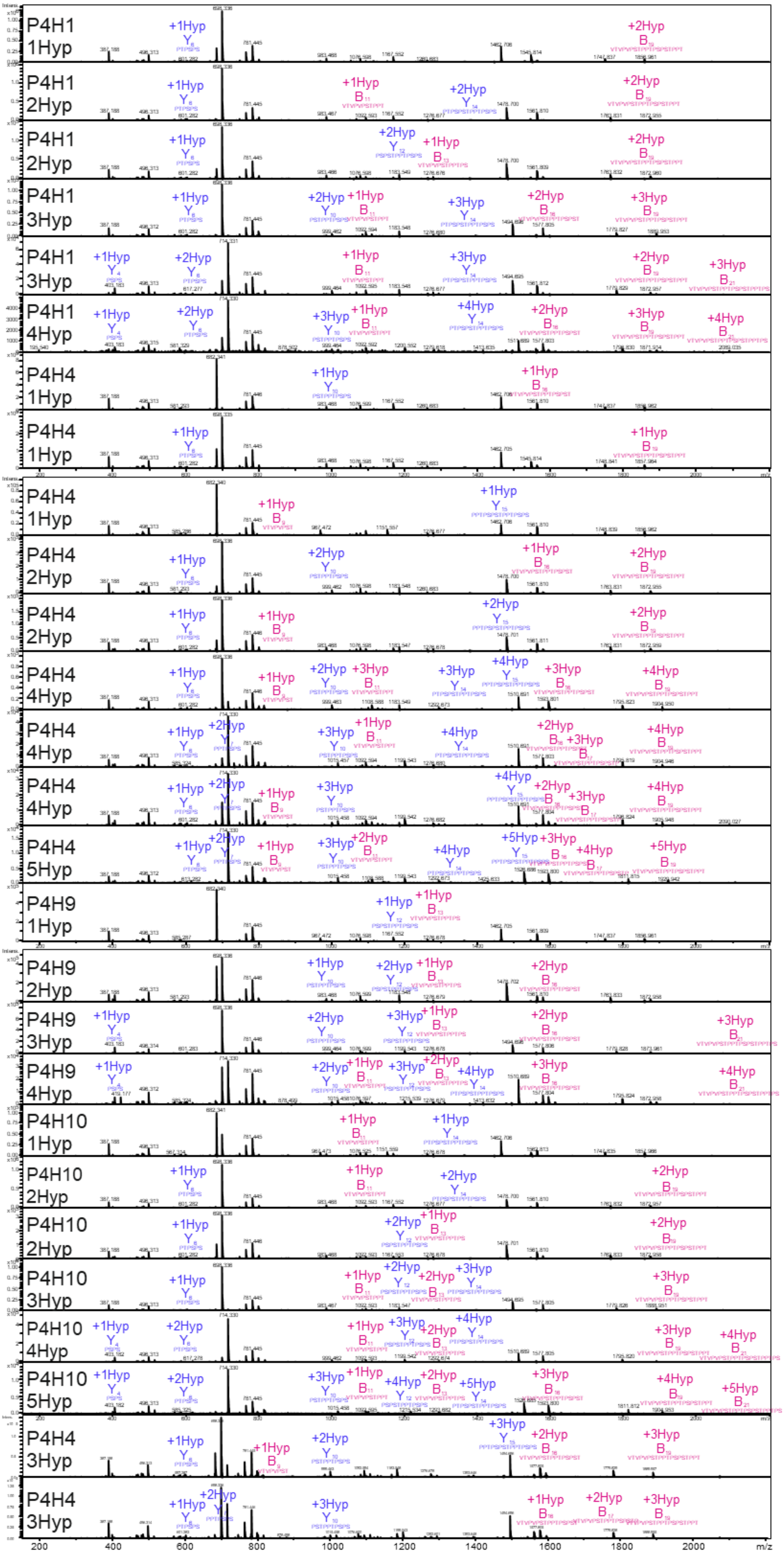
P4H1 1Hyp
P4H1 2Hyp
P4H1 2Hyp
P4H1 3Hyp
P4H1 3Hyp
P4H1 4Hyp
P4H4 1Hyp
P4H4 1Hyp
P4H4 1Hyp
P4H4 2Hyp
P4H4 2Hyp
P4H4 4Hyp
P4H4 4Hyp
P4H4 4Hyp
P4H4 5Hyp
P4H9 1Hyp
P4H9 2Hyp
P4H9 3Hyp
P4H9 4Hyp
P4H10 1Hyp
P4H10 2Hyp
P4H10 2Hyp
P4H10 3Hyp
P4H10 4Hyp
P4H10 5Hyp
P4H4 3Hyp
P4H4 3Hyp
200
400
600
800
1000
1200
1400
1600
1800
2000
m/z

Supplementary Figure S5. Individual LC-ESI-MS/MS spectra of the separate Base Peak Chromatogram peaks of **Figure 4** used to determine oxidation sites. Diagnostic Y-ions are marked blue and B-ions pink. Substrate was a synthetically produced IgA1 peptide with the sequence of VTVPVPSTPPTPSPSTPPTPSPS.

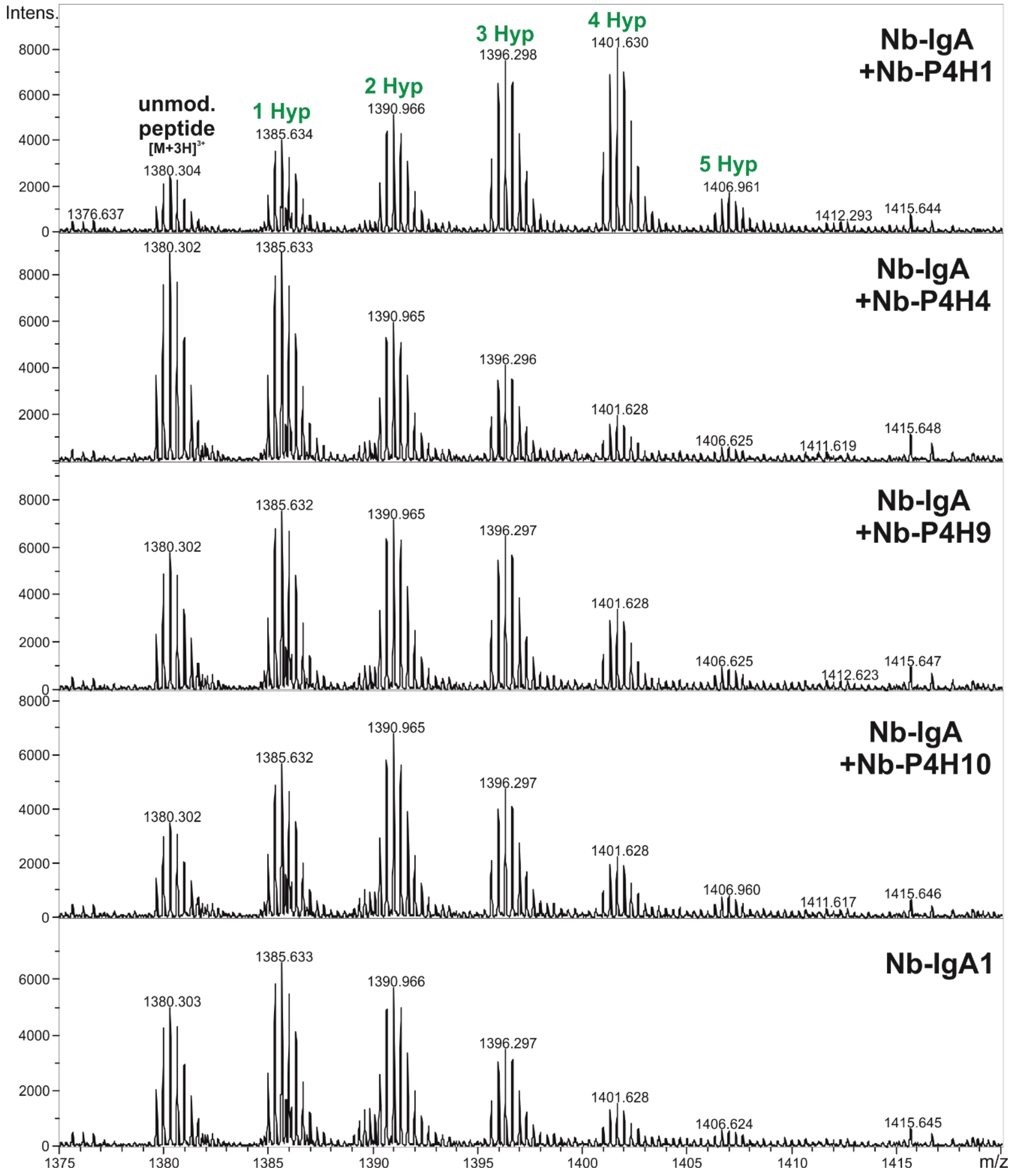

Supplementary Figure S6. LC-ESI-MS spectra of the IgA1 tryptic peptide (HYTNPSQDVTVPCPVPSTPPTPSPSTPPTPSPSCCHPR - 4136.8899 Da) obtained after expressing IgA1 together with overexpression constructs of P4H candidates in *Nicotiana benthamiana*. After overexpression, a maximum of 6 hydroxyproline residues were attached to the peptide. Relative quantification of the data can be found in **Table 2** of the manuscript.

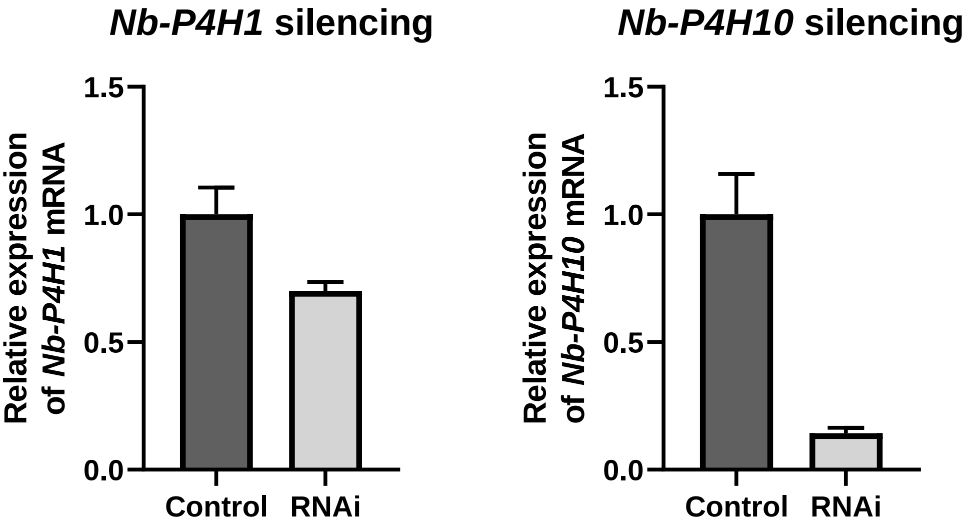

Supplementary Figure S7. Relative transcript levels of *Nb-P4H1* or *Nb-P4H10* mRNA in wild-type *N. benthamiana* leaves infiltrated with a control or a Nb-P4H gene silencing construct. Expression levels were analyzed by RT-qPCR. Values and error bars indicate means ± SD (n = 3).

SUPPLEMENTARY DATA

Supplementary Data S1. ORF regions of the selected *Nb-P4H* candidates amplified using *N. benthamiana* cDNA library and subcloned into pMiniT 2.0 vectors (NEB PCR mini kit; New England Biolabs, Frankfurt am Main, Germany). The sequences have been submitted to the NCBI nucleotide database and the GenBank accession numbers are in brackets.

pMiniT 2.0-*Nb-P4H1* (GenBank: MW524054)

ATGGCTTCGGCAATGAGAATTGTTTTCGGGCTTCTTACACTTGTCACTTGTGGAATGATTCTCGGAGC
TTTGATTCAACTAGCATTTATTCATAGGATGGAGGACTCACTTGACAGAGAATCGTCTTTTGGTGGAA
AGCATGCAAGTTTAGCTGGCAACAAACATCCGTTAGCAAGAGGAATCTCTTATTGGGGTTACGACAAA
GAAGCTGTAGCACTACGTATAGGATATGTGAAGCCTGAAATTATTAGCTGGAAACCAAGAATTATATT
ATTTCACAACTTCTTAAGTGCAGAGGAATGTGATTATCTTAGATCGGTTGCCATGCCCCGTCTTCATG
TTTCAACTGTTGTAGATGCAAAAACTGGAAAGGGAATTAAGAGTGATGTCAGAACAAGTTCGGGAATG
TTTTTGAGCCCTGATGAGAGGAAGTATCCCATGATACAGGCAATTGAAAAACGAATTTCTGTATATTC
TCAAATACCAGTTGAAAATGGGGAACTCATTCAAGTGTTAAGGTATGAAAAGAATCAGTTCTATAGAG
CCCATCACGACTATTTCTCTGATTCATTTAATGTGAAGCGTGGAGGTCAACGAATAGCAACAATGCTC
ATGTATTTGAGCGACGGCGTTGAGGGGGGAGAGACATACTTTCCCATGGCTGGCACTGGTGAATGTAG
CTGTGGTGGCAAAATGATCAAAGGGTTATGTGTAAAACCTACTAAAGGAGATGCTGTTCTTTTTTGGA
GCATGGGGCTTGATGGACAATCTGACCCTGAGAGTTTACATGGAGGATGTGAAGTACTCTCAGGAGAG
AAGTGGTCAGCTACTAAATGGATGAGGCAAAGACTTGTATCCTAA

pMiniT 2.0-*Nb-P4H4* (GenBank: MW524055)

ATGAACAGCTCTTTGCTGCTCGCTACTTTCTTCTTCCTCTTCATCATAGCTTTTGTGCGCGAATCGTC
CAGCTCAGCGATCATCAACCCATCCAAAGTCAAGCAAATTTCATGGAAACCTAGAGCATTTGTATATG
AAGGATTTCTTACGGATGAAGAATGCAATCACTTGATATCTCTCGCAAAATCGGAGCTGAAGAGATCG
GCTGTGGCAGACAATGAGTCTGGAAATAGTAAGACCAGTGAGGTTAGGACTAGCTCCGGAATGTTCAT
TCCCAAAGCTAAGGATCCTATTGTTTCTGGGATAGAGGAGAAGATAGCAACTTGGACTTTTCTACCAA
AAGAGAATGGAGAAGAAATACAAGTACTAAGATATGAGGAGGGGCAGAAATATGAGCCACACTATGAT
TACTTTGTAGACGAGGTTAATATTGCTCGAGGTGGACATCGCTTGGCCACTGTACTTATGTATCTCAC
AGATGTTGAAAAGGGAGGTGAAACTGTCTTCCCTAATGCAGAGGAATCACCTCGGCGTAGGTCGATGA
CAGCAGATGACAGCTTGTCTGAATGTGCAAAGAAGGGTATACCAGTGAAACCACGGAAAGGAGATGCC
CTTCTTTTCTACAGTCTACATCCAAATGCCACTCCTGATCCTCTTAGTCTCCATGGTGGGTGCCCTGT
CATTCAAGGTGAGAAATGGTCAGCAACAAAGTGGATTCATGTGGATTCCTTTGTCAAAACTGTGGAGA
CCGTAGGAAATTGCAGCGATCGTGATGAGAATTGTGAGAGATGGGCTGCTCTTGGGGAATGCACCAAG
AATCCAGAGTACATGCTGGGAAGTGCAGGCCTTCCAGGATATTGTAGGAAGAGCTGCAAAGCCTGTTA
A

pMiniT 2.0-*Nb-P4H9* (GenBank: MW524056)

ATGAAGAACAGAGGCAAATTACCGGGACAAAGATGGTGGAGCTTAGGGTTACCTTCGGTTTTTCTCCT
TTGTCTTTTCTTCTTCCTCGTCGGTTTATTCGGTTCTACTTTCATCTCTCAGCAGGATGTACAAGTAG
GTAGTGTACGACCTAGGTCGAGGGTGCTTGAATCTGTGGAAGAATTTGATGCTTTGCCCAATGGCGAG
ACTGGAGAACATTCACTCACTTCCATCCCCTTTCAGGTCTTAAGCTGGTTCCCGCGTGCATTATACTT
TCCCAATTTTGCAACTGAAGAACAATGCCAAGGCATTATTAAGATGGCAAAGGCAGAGCTGAAACCAT
CAGCTTTGGCTCTCCGCAAAGGAGAAACAGCAGAGAATACCAAAGGAATAAGAACAAGTTCTGGAATG
TTTATCAGTTCATCTGAAGACAAAACTGGAATTTTGGACCTCATTGAGGAAAAAATTGCAAGGGCGGC
TATGATCCCCAGGACACATGGAGAGGCATTTAATGTGTTGCGGTATGAAATCGGCCAGAGTTATCATT
CACATTATGATGCATTTGATCCTTCTCAATATGGTCCTCAGAAGAGCCAAAGGGTTGCATCCTTTTTA
TTGTATTTATCTGATGTGGAAGAAGGTGGAGAGACCATGTTTCCTTTCGAGAATGGGCAGAACATGGA
TGCTAATTATGACTTCCGAAAGTGTATTGGTTTGAAAGTGAAGCCGCGTAGAGGGGATGGACTACTGT
TCTACTCACTGTTTCCAAATGGTACAATTGATCCGACATCTCTTCACGGGAGCTGTCCAGTAATCAGA
GGCGAAAAATGGGTTGCCACAAAGTGGATCAGGGATCAGGAACTTGATGAATAA

pMiniT 2.0-*Nb-P4H10* (GenBank: MW524057)

ATGGCAGTCAAAGGAAGGCACGTCCGAGGTGGTCTACCTCGTAAATCATCGAATTCGACGCTGGTCTT
TGCCGTATTTATTGGATTATCTCTTCTCGTTTTGATTCTTCTCGCTTTTGGAATTTTCTCGATTCCTT
TCAGTTCTAAAGGATCTCAAAAAGCACATGATCTTAGCTCAATTGCGCACAACGCAGTTGGAAGACGA
GGTGATGATGGTGGAAAAGGAGATCAGTGGGCTGAAGTGATTTCATGGGAACCAAGAGCTGTTGTGTA
CCATAACTTCTTGTCAAAGGACGAATGTGAATATCTGATTAATCTTGGCAAGCCTCATATGAAAAAGT
CAACTGTCGTCGACAGTGCTACTGGCAAGAGTACTGATAGCAGGGTTCGAACAAGTTCTGGAACATTT
CTTGCCAGGGGACAAGATAAAGTAGTCAGGACTATAGAGAAAAGGATTGCAGATTTTACTTTTATACC
AGTAGAGCATGGTGAAGGTCTACAAATTCTCCACTATGAAGTTGGGCAAAAGTATGAGCCACACTATG
ATTACTTTGCCGAAGAGTTCAATACTATAAATGGTGGTCAACGCATTGCCACGGTTTTGATGTACTTA
TCAGATGTGGAAGAAGGGGGAGAAACTGTATTTCCTACTGCCAAGGGAAATGTTAGTGCGGTTCCTTG
GTGGAATGAGCTATCTGAATGTGGAAAAGGTGGACTCTCTGTAAAACCAAAGATGGGTGATGCTTTGC
TTTTCTGGAGCATGAAGCCTGATGCTACTCTTGATCCTTCAAGCTTGCATGGTGGGTGCCCTGTGATT
AAGGGTAACAAGTGGTCGTCTACGAAATGGATGCGTGTTCACGAATACAAGGTTTAA

Supplementary Data S2. Sequence A: Synthetic *NbP4H1*-RNAi sequence in pMA-GeneArt cloning vector. The sequence consists of a *N. benthamiana P4H1* cDNA fragment (bold), restriction enzyme cleavage sites (underlined) and the intron 2 (italics) from the *Arabidopsis thaliana* β1,2-xylosyltransferase gene (At5g55500) (Strasser et al., 2008). **Sequence B:** Synthetic *NbP4H10*-RNAi sequence in pMA-GeneArt cloning vector. The sequence consists of a *N. benthamiana P4H10* cDNA fragment (bold), restriction enzyme cleavage sites (underlined) and the intron 2 (italics) from the *Arabidopsis thaliana* β1,2-xylosyltransferase gene (AT5g55500) (Strasser et al., 2008).

Sequence A:

TTCTA**GAGGGGGGAGAGACATACTTTCCCATGGCTGGCACTGGTGAATGTAGCTGTGGTGGCAAAATG**
ATCAAAGGGTTATGTGTAAAACCTACTAAAGGAGATGCTGTTCTTTTTTGGAGCATGGGGCTTGATGG
ACAATCTGACCCTGAGAGTTTACATGGAGGATGTGAAGTACTCTCAGGAGAGAAGTGGTCAGCTACTA
AATGGATGAGGCAAGATCT*GTATGCTCCTCTTCTTGTTCATGGTCATGATCCTTATATGAGCAGGGAA*
AGTCCAGTTTAGACTTGTAGTTAGTTACTCTTCGTTATAGGATTTGGATTTCTTGCGTGTTTATGGTT
TTAGTTTCCCTCCTTTGATGAATAAAATTGAATCTTGTATGAGTTTCATATCCATGTTGTGAATCTTT
*TTGCAG*GGTACCTTTGGATCC

Sequence B:

aaTCTAGA**AAATGGTGGTCAACGCATTGCCACGGTTTTGATGTACTTATCAGATGTGGAAGAAGGGGG**
AGAAACTGTATTTCCTACTGCCAAGGGAAATGTTAGTGCGGTTCCTTGGTGGAATGAGCTATCTGAAT
GTGGAAAAGGTGGACTCTCTGTAAAACCAAAGATGGGTGATGCTTTGCTTTTCTGGAGCATGAAGCCT
GATGCTACTCTTGATCCTTCAAGCTTGCATGGTGGGTGCCCTGTGATTAAGGGTAACAAGTGGTCGTC
TACGAAATGGATGCAGATCT*GTATGCTCCTCTTCTTGTTCATGGTCATGATCCTTATATGAGCAGGGA*
AAGTCCAGTTTAGACTTGTAGTTAGTTACTCTTCGTTATAGGATTTGGATTTCTTGCGTGTTTATGGT
TTTAGTTTCCCTCCTTTGATGAATAAAATTGAATCTTGTATGAGTTTCATATCCATGTTGTGAATCTT
*TTTGCAG*GGTACCTTTGGATCC
